# A multimodal dataset for investigating working memory in presence of music: a pilot study

**DOI:** 10.3389/fnins.2024.1406814

**Published:** 2024-06-19

**Authors:** Saman Khazaei, Srinidhi Parshi, Samiul Alam, Md. Rafiul Amin, Rose T. Faghih

**Affiliations:** ^1^Department of Biomedical Engineering, New York University, New York, NY, United States; ^2^Department of Electrical and Computer Engineering, University of Houston, Houston, TX, United States

**Keywords:** multimodal dataset, brain-machine interface (BMI), music, closed-loop systems, working memory, decoder design, cognitive arousal, cognitive performance

## Abstract

**Introduction:**

Decoding an individual's hidden brain states in responses to musical stimuli under various cognitive loads can unleash the potential of developing a non-invasive closed-loop brain-machine interface (CLBMI). To perform a pilot study and investigate the brain response in the context of CLBMI, we collect multimodal physiological signals and behavioral data within the working memory experiment in the presence of personalized musical stimuli.

**Methods:**

Participants perform a working memory experiment called the *n*-back task in the presence of calming music and exciting music. Utilizing the skin conductance signal and behavioral data, we decode the brain's cognitive arousal and performance states, respectively. We determine the association of oxygenated hemoglobin (HbO) data with performance state. Furthermore, we evaluate the total hemoglobin (HbT) signal energy over each music session.

**Results:**

A relatively low arousal variation was observed with respect to task difficulty, while the arousal baseline changes considerably with respect to the type of music. Overall, the performance index is enhanced within the exciting session. The highest positive correlation between the HbO concentration and performance was observed within the higher cognitive loads (3-back task) for all of the participants. Also, the HbT signal energy peak occurs within the exciting session.

**Discussion:**

Findings may underline the potential of using music as an intervention to regulate the brain cognitive states. Additionally, the experiment provides a diverse array of data encompassing multiple physiological signals that can be used in the brain state decoder paradigm to shed light on the human-in-the-loop experiments and understand the network-level mechanisms of auditory stimulation.

## 1 Introduction

Recent advances in physiological signal measurement techniques enlighten the non-invasive closed-loop brain-machine interface (BMI) design. Such signals can be used to infer individuals' underlying cognitive brain states and regulate them via non-invasive interventions such as music (Fekri Azgomi et al., 2023). The human brain state regulation via musical stimuli would have profound applications in closed-loop BMI (CLBMI) (Ehrlich et al., 2019), neural rehabilitation (Ottonello et al., 2019; Salas et al., 2019), and cognitive impairment treatments (Ray and Mittelman, 2017). As an instance of such applications, the well-known Yerkes-Dodson law, a.k.a. the inverted-U law, explains that optimal cognitive performance can be achieved by setting the cognitive arousal within a moderate level (Yerkes, 1907; Yerkes and Dodson, 1908). This inspires us to design a pilot study involving a human-subject working memory experiment in the presence of arousing music stimuli. We record the multimodal physiological and behavioral signals to investigate the feasibility of regulating one's cognitive arousal and performance via background music with calming and exciting contents.

Several studies have explored how the introduction of music can impact cognitive functioning (Parshi et al., 2019; Khazaei et al., 2021). Multiple studies have used music to influence driving performance (Ünal et al., 2012, 2013). In Huang and Shih (2011) and Kuschpel et al. (2015), the positive effect of music on concentration and the effectiveness of using music to reduce cognitive stress in closed-loop systems have been shown. In our designed working memory experiment, two types of music are employed, namely, calming and exciting music. Notably, the calming and exciting music selected by participants such that the calming and exciting components are supposed to replicate the low and high arousing conditions, respectively. Here, we mainly focus on cognitive performance and arousal in the presence of calming and exciting music.

Cognitive performance is a hidden brain state that demonstrates the general performance of one's cognitive functions (Khazaei et al., 2024). The cognitive functions of humans can be divided into two groups, namely, basic cognitive functions and higher-level cognitive functions. Working memory is one of the basic cognitive functions that provides temporary storage and allows the manipulation of information (Baddeley, 1992). Here, the *n*-back task serves as a cognitive task of interest, which encourages working memory usage by inducing different cognitive loads (von Janczewski et al., 2021; Fekri Azgomi et al., 2023). Decoding the underlying cognitive performance in a continuous manner is one of the challenges in this paradigm, which can be addressed by employing informative data and applying decoding approaches. One of the most accessible and informative data in this context would be the behavioral data recorded during the cognitive task of interest. We consider the sequence of responses as well as the reaction time of participants as the available behavioral observation and quantify the cognitive performance using Bayesian filters within an expectation-maximization (EM) framework (Amin et al., 2021; Khazaei et al., 2021).

Another hidden brain state that may have impact on cognitive performance is the underlying arousal state. In particular, the amygdala plays a crucial role in connecting arousal to memory formation (McGaugh, 2004). Typically, the arousal state is linked to the degree of physiological alertness (Cudo et al., 2018), and variation in arousal is mainly due to the exogenous and endogenous stimulation, which can be accompanied by neural, hormonal, and other biochemical changes (Hobson and Lindsley, 1988). Specifically, previous research on the autonomic nervous system (ANS) inference presents the electrodermal activity (EDA) as an informative measurement of cognitive arousal (Wickramasuriya et al., 2018; Wickramasuriya and Faghih, 2020a). In particular, the skin conductance (SC) signal (a measure of EDA) reflects the sweat secretions process, which is firmly linked to the underlying ANS activity inside the brain (Amin and Faghih, 2022). The variation in skin conductivity can be used as a quantitative index of arousal during a cognitive task (Khazaei et al., 2021).

When it comes to designing a CLBMI architecture for human cognitive functions, it would be crucial to investigate how the environment and interactions affect the cognitive capacity of the human operator (Cain, 2007). To gain a better insight into BMI design procedures and analyze how different regions of the brain react to the stimuli, neuroimaging methods with relatively high spatial resolution can play a crucial role. These neuroimaging methods include but are not limited to magnetoencephalography (MEG), electroencephalography (EEG), functional magnetic resonance imaging (fMRI), and functional near-infrared spectroscopy (fNIRS) (Berka et al., 2007; Wendel et al., 2009; Baldwin and Penaranda, 2012; Fyshe et al., 2012). The fNIRS is a relatively new neuroimaging technique that can show significant spatiotemporal changes for memory tasks in the prefrontal cortex (PFC) (Hoshi et al., 2003). Also, the fNIRS has a higher spatial resolution than EEG, and it is practical for unobtrusive applications. In the designed experiment, we use the fNIRS and collect data from the prefrontal cortex (PFC) and occipital (OC) areas of the participant.

Multiple studies investigate the brain neural activity in the course of *n*-back tasks via fNIRS (Ayaz et al., 2007; Roy et al., 2013; Fishburn et al., 2014; Herff et al., 2014). In Ayaz et al. (2007) and Herff et al. (2014), the cognitive loads and hemodynamic responses association is considered, and the hemodynamic responses are classified accordingly. Another study considers the *n*-back tasks and presents the fNIRS sensitivity to cognitive load and transitioning from a resting state to a task (Fishburn et al., 2014). While the link between the cognitive load of *n*-back task and corresponding hemodynamic responses is studied vastly, the study of association between the continuous cognitive performance signal and fNIRS data in the presence of music is investigated relatively sparsely. Previous studies such as Meidenbauer et al. (2021) and Struckmann et al. (2021) have considered the distinctive measures of cognitive performance, like the number of correct/incorrect responses and reaction time, and investigated the hemodynamic response data association. While this set of measurements can be an informative index of performance, it merely presents a distinct measure of performance, and might not fully capture the underlying dynamic of the brain's cognitive state (Basu et al., 2023). In this research, we study the association between the oxygenated hemoglobin (HbO) concentration and the continuous performance state decoded from behavioral data. In particular, we employ a Bayesian-based performance decoder that accounts for the underlying dynamic of this brain state, and tracks its continuous trajectory using behavioral data. Also, we evaluate the energy of total hemoglobin (HbT) signal within each music session.

To summarize, the primary purpose of this pilot study is to provide a multimodal database and examine the viability of using music as an intervention. The database includes a diverse array of data encompassing multiple physiological measurements to shed light on the human-subject experiments using music intervention. The available signals include SC data, electrocardiogram (ECG), skin surface temperature, respiration, photoplethysmography (PPG), functional near-infrared spectroscopy (fNIRS), electromyogram (EMG), de-identified facial expression scores, sequence of correct/incorrect responses, and reaction time recorded during the *n*-back task. In this research, we focus on cognitive performance and arousal, and we present how the HbO concentration is correlated with cognitive performance. Also, the energy of the HbT signal is evaluated with respect to each music. We represent how the arousal and performance indices vary with respect to task difficulty and music type. Then, we discuss our findings and provide a summary of our approach, followed by future directions of this research.

## 2 Materials and methods

### 2.1 Cognitive task overview

We designed the experiment centered on the working memory task called the *n*-back task (Herff et al., 2014; Shin et al., 2018). Here, the participant was shown a series of alphabets as stimuli, and the participant had to identify if the most recently displayed alphabet was the same as the alphabet displayed at the *n*^*th*^ previous iteration (Herff et al., 2014; Shin et al., 2018; Khazaei et al., 2021). In this type of experiment, the cognitive workload increases with *n*, and the participant has to recall more of the stimuli with higher values of *n*. Here, the experiment was conducted within two sessions; session one was accompanied by calming background music, and session two was accompanied by exciting background music. The participants were requested to share their own music with “calming” and “exciting” content to be played during the experiment. We employ this strategy first to enhance the ecological validity, as people often listen to their own music (Ünal et al., 2012); secondly, this strategy aims to ensure that any behavior observed in brain activity is not attributable to disliking the music (Ünal et al., 2012). Finally, this strategy enables us to incorporate the person-specific closed-loop system with personalized intervention. We delivered the background music via an external speaker. The music stimulation started at the beginning of each session, and it continuously played until the end of the session (i.e., 964 s). We performed the experiment in a closed experiment room to minimize the impact of external variables. Each session in the experiment included 8 blocks for each type of *n*-back task (2 types of *n*-back task × 8 = 16 blocks within one session). Hence, we have 16 blocks at each session and 32 blocks during the whole experiment. We decided to keep the experimental duration time for a bit longer than half an hour. This ensures that the collected SC signals do not show a lot of relative drift over time due to the accumulation of sweat or shift in the position of electrodes.

**Figure 1:**
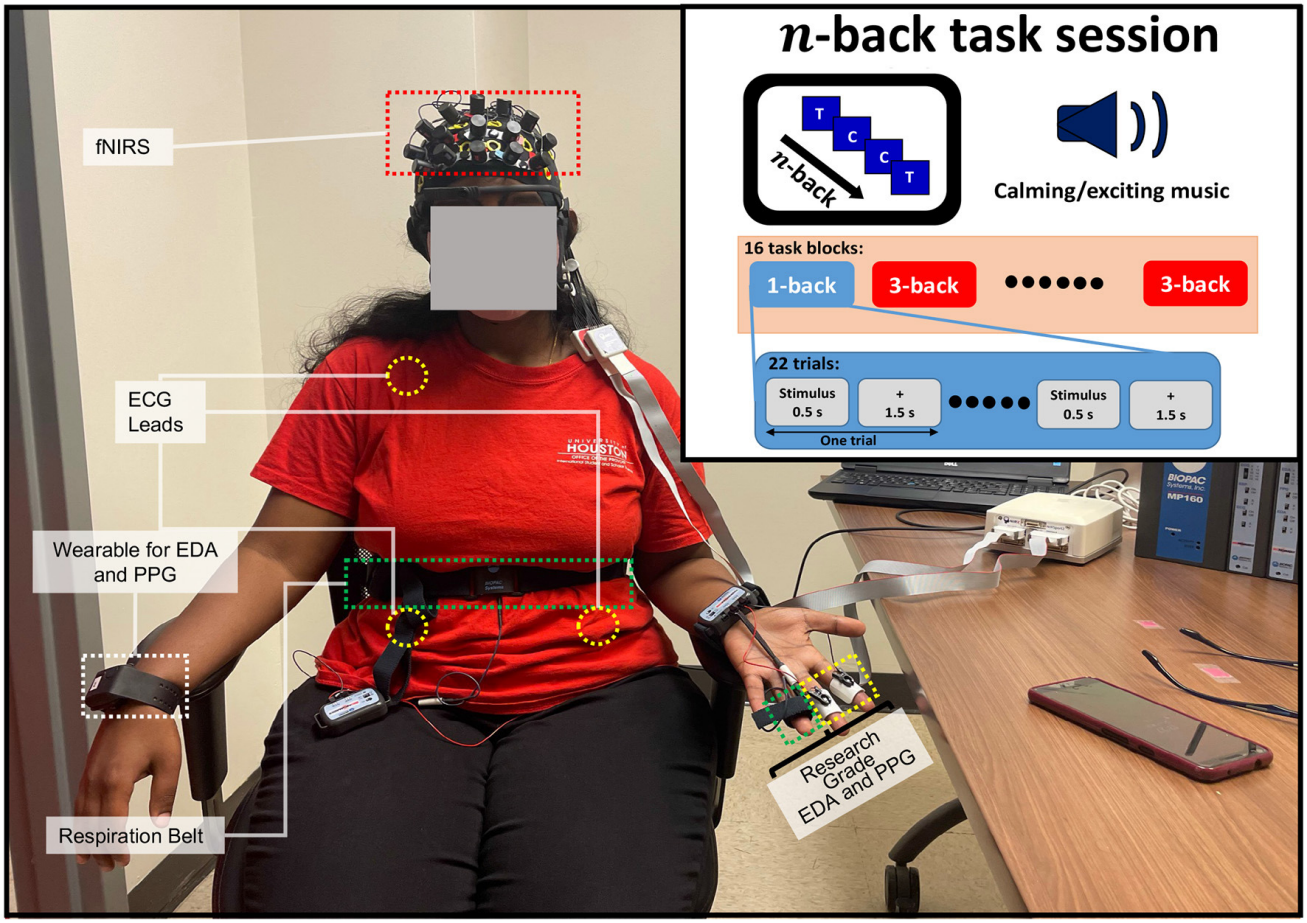
The experimental setup for multimodal physiological data collection from a female participant during the *n*-back task. An overview of the physiological sensors' placement during the *n*-back task session.

Each block includes 5 s instruction followed by 22 trial windows in which a letter stimulus was displayed for 0.5 s and a cross was presented for 1.5 s, which resulted in a total 2 s trial window that participant could deliver the response via Chronos Keypad. Thus, the total duration of each task block was 49 s (5+22 × 2 = 49). In each task block, 30% of the stimuli were a target. The task block of each *n-*back task was randomized. At the end of each block, a 10 s “RELAX” segment was presented where a resting cross was displayed on the screen. After 8 task blocks (the halfway mark for each session), a 20 s “RELAX” section was presented where a resting cross is displayed on a smart 65 inch TV screen connected via HDMI to a laptop. The duration of each session was 964 s, which is ~16 min. After each session, there was a 2 min relaxation break in which the participant was allowed to relax. A resting cross was displayed on the screen during this time. Figure 1 describes the timing of one session with randomized trials. The experiment took a total of 2, 168 s (calming session duration 964+ intersession break 120+ exciting session duration 964+ after the session rest period 120 = 2, 168 s), i.e., approximately half an hour. Participants were comfortably seated with the attached non-invasive sensors, and a display screen was placed ~1–2 m in front of them. The only required movement was pressing one of two buttons on a Chronos Keypad: the target and non-target buttons. Participants were required to press one of the buttons for each stimulus displayed.

### 2.2 Experimental procedures

The experimental procedures in this study were approved by the institutional review board at the University of Houston, TX, USA (STUDY00002013). Only participants who were 18 years or older and were able to provide consent were permitted to participate in this experiment. Anyone suffering from known cardiac ailments or psychological disorders was excluded. Adults unable to consent, anyone below age 18, pregnant women, prisoners, students whose grades may be influenced and, economically and/or educationally disadvantaged persons were excluded from the study. A total number of 11 healthy participants (five male and six female) between the ages of 22–25 participated in this study. Six participants with measurement error, data corruption, and small modalities were removed. Hence, the studied sample size here is five (two male and three female) while the de-identified facial expression scores for four participants are included in the main database. All identifiable aspects of the data were removed to ensure privacy. This includes any data that may be used to identify the original participants. The experiment focused on collecting multimodal data to investigate the feasibility of using music as an intervention. The experiment was conducted with a multitude of sensors. We describe the sensors applied in the following subsections.

#### 2.2.1 Functional near infrared spectroscopy

The near-infrared region (620–1, 000 nm) of the electromagnetic spectrum is scattered by biological tissue but absorbed by hemoglobin (Villringer et al., 1993); by measuring the amount of absorbed near-infrared light and using the modified Beer-Lambert law (Sassaroli and Fantini, 2004), fNIRS measures changes in oxygenated hemoglobin (HbO), deoxygenated hemoglobin (HbR), and total hemoglobin (HbT). The fNIRS demonstrates excellent spatial resolution but relatively poor temporal resolution (Fazli et al., 2012). The spatial resolution of fNIRS can be used to obtain the functional connectivity map of the brain (Santosa et al., 2018). The fNIRS optodes can be placed according to the international 10 − 5 system (Oostenveld and Praamstra, 2001), and readings can be taken from the whole scalp. The fNIRS channels placed during this experiment collected hemodynamic data from the PFC and the OC areas of the brain. In particular, the employed fNIRS sensor is *NIRSport 2*, configured as shown in Figure 4. The sources (S) and detectors (D) were placed according to the positions depicted in the figure (on a head cap worn by the participants). There were 16 sources and 14 detectors located on PFC and OC areas, and signals were recorded from 44 channels. The sampling frequency is 7.81 Hz.

#### 2.2.2 Electrocardiogram

**Figure 2:**
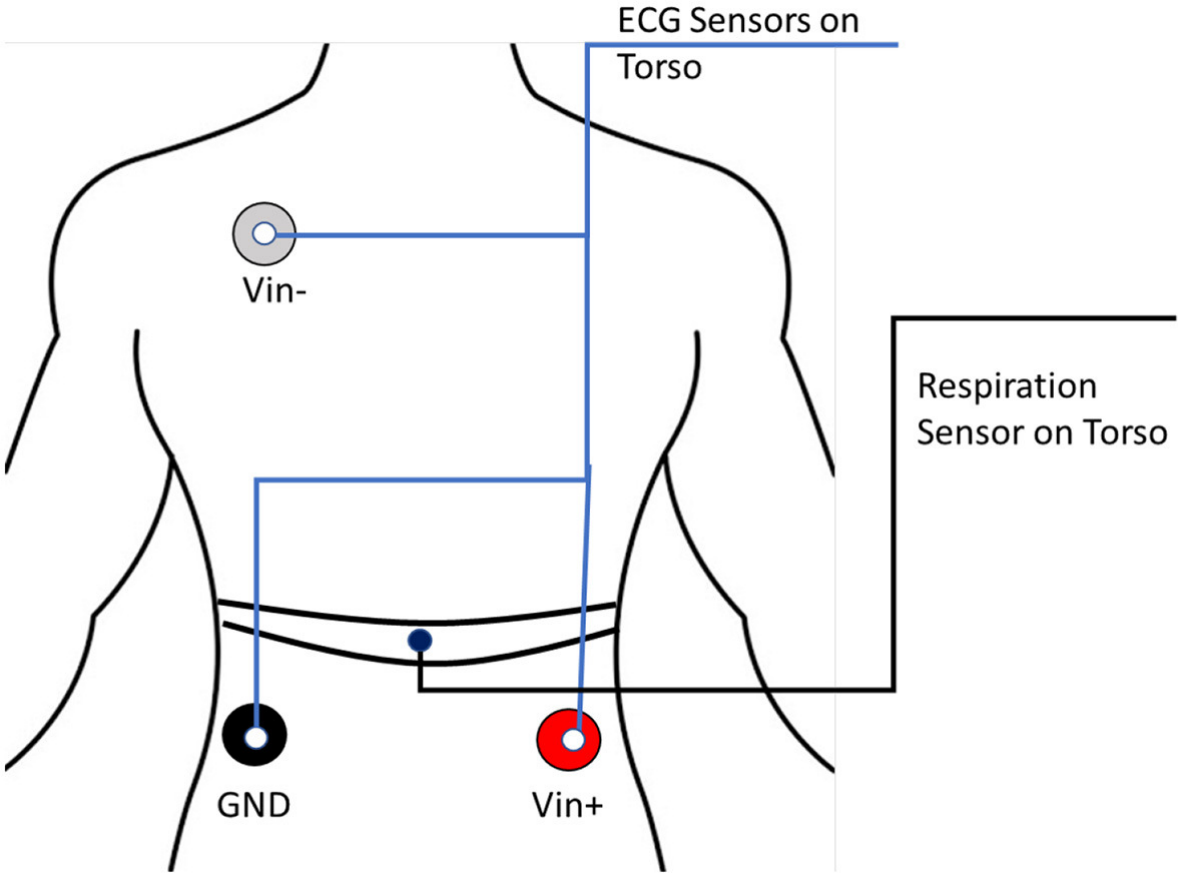
The electrocardiogram (ECG) and respiration sensors configuration. Electrode placements of Electrocardiogram sensors (indicated by blue lines) and respiration belt on the front torso (shown with black lined indicators).

ECG is the electrogram of the heart. Specifically, it is the electrical signal that correlates with the expansion and contraction of the heart muscle, and it is used to detect heart problems such as arrhythmia. In our experiment, ECG sensors were placed on the torso of the participants, as shown in Figure 2. We collected the ECG data with the *MP160 BioPac* system and the *BioNomadix* wireless devices. The *EL503 BioPac* general-purpose disposable electrodes were used on the torso region. The sampling frequency is 2, 000 Hz.

#### 2.2.3 Respiration

The respiration belt sensor of the *MP160 BioPac* system was placed on the abdomen of the participant in contact with the torso as described in the *BioPac* manual and depicted in Figure 2. The contraction and expansion of the lungs are captured by the belt. The sampling frequency is 2, 000 Hz.

#### 2.2.4 Skin surface temperature

**Figure 3:**
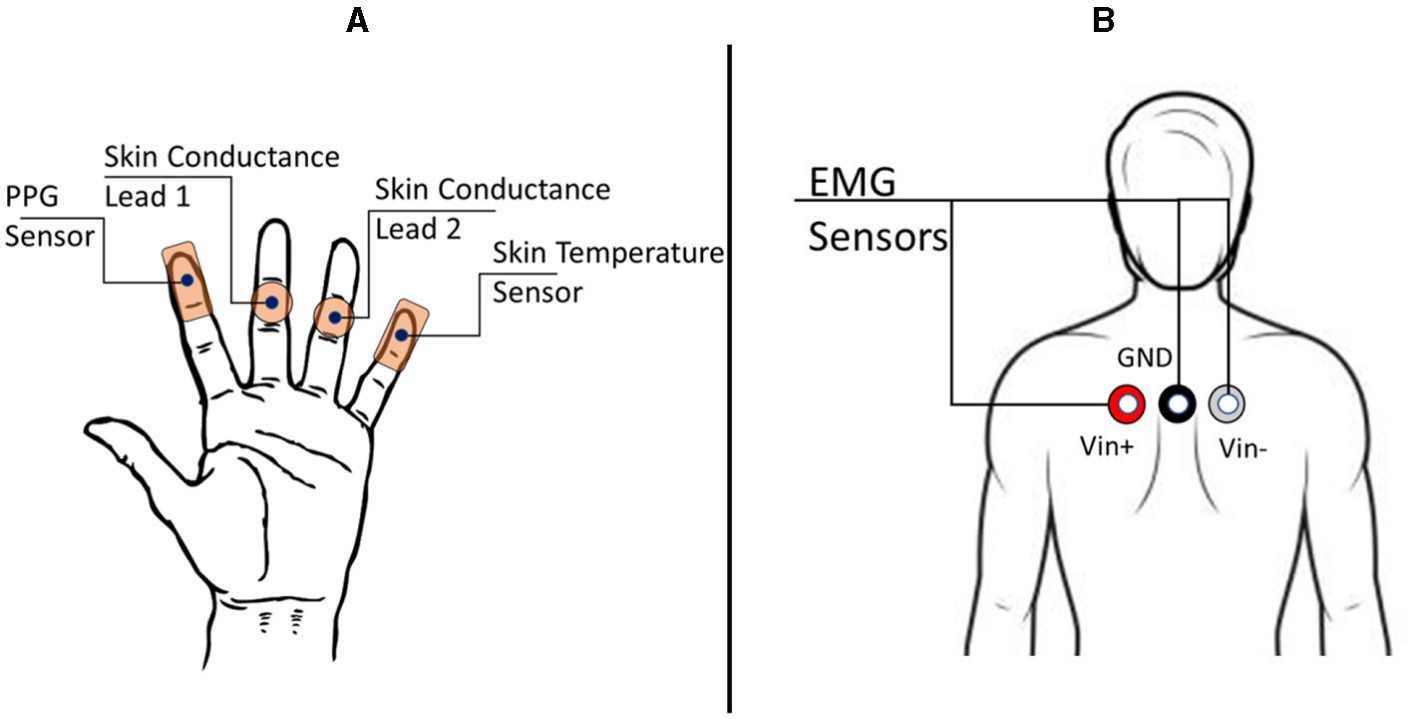
The electrodermal activity (EDA), photoplethysmography (PPG), and electromyogram (EMG) sensors configuration. **(A)** Sensor placements on the hand for PPG, EDA, and Skin Temperature. **(B)** EMG sensors were placed on the participant's trapezius muscles to detect stress states from muscle movements.

As portrayed in Figure 3, the skin surface temperature data is collected from the minimus digitus (little finger) of the non-dominant hand using the MP160 BioPac system with the *BioNomadix* wearable device coupled with the *BN-TEMP-A-XDCR BioPac* sensor. Also, the *Empatica E4* wearable wristband worn by the participant collected skin temperature data. The sampling frequency for BioPac is 2, 000 Hz, and for *Empatica E4* is 4 Hz.

#### 2.2.5 Electrodermal activity

Sensors from both the *MP160 BioPac* system and *Empatica E4* wearable wristband were used to record EDA. The *Empatica E4* wearable wristband was worn on the wrist by the participant. The *M160 BioPac* system sensors were placed over the digitus quartus manus (ring finger) and digitus medius manus (middle finger) of the participant, as shown in Figure 3. The BioPac EL507 disposable electrodes are used as the leads for EDA. The sampling frequency for *BioPac* is 2, 000 Hz, and for *Empatica E4* is 4 Hz.

#### 2.2.6 Photoplethysmography

Wearable physiological sensor *BN-PULSE-XDCR* coupled with *BioNomadix* unit is placed on the digitus secundus manus (index finger) of the non-dominant hand (Figure 3) to obtain PPG data with the *M160 BioPac* system. Also, the *Empatica E4* wearable wristband (worn on the wrist) collected PPG data. PPG is an optical means to detect changes in blood volume in a tissue. PPG is generally used to monitor cardiac health and heart rate. The sampling frequency for the *BioPac* system is 2, 000 Hz. The sampling frequency for the *Empatica E4* wristband is 4 Hz.

#### 2.2.7 Electromyogram

As depicted in Figure 3, sensors from the *MP160 BioPac* system are placed on the participant's trapezius muscle for EMG recordings. The *EL503* general-purpose electrodes are used in this case. EMG is used to detect the health of muscles and the nerves that control them. In this experiment, the placement of EMG electrodes provides data about the tensing of a participant's shoulders and back while performing a cognitive stress task. The sampling frequency is 2, 000 Hz.

#### 2.2.8 Facereader

Facial expression data were recorded via a dedicated camera. Then, the facial expression scores were obtained using Face Reader software. De-identified facial expression scores for four participants are included in the multimodal dataset.

#### 2.2.9 Synchronization and behavioral data recording

The experimental design, timing, and triggers for different equipment have been executed using the *Chronos* input device and *E-Prime* software. The *Chronos* and *E-Prime* offer a script-free way to synchronize data with task events. The participant's behavioral signals, including the number of correct and incorrect responses along with reaction times, were collected.

### 2.3 Data analysis methods

To decode the cognitive state of interest, we employ the Bayesian filtering approach within the expectation-maximization framework. We utilize the SC signal collected via MP160 BioPac system to decode a cognitive arousal state. This is done by considering the arousal events occurrences and their amplitudes as the available observation (Wickramasuriya et al., 2018, 2019, 2022; Wickramasuriya and Faghih, 2019a,b, 2020a,b). Also, to quantify the hidden performance state, we use the sequence of correct and incorrect responses and the reaction time as binary and continuous observations, respectively (Prerau et al., 2009).

#### 2.3.1 Arousal state estimation

The arousal state (*x*_*j*_) can be modeled as a random walk process (Wickramasuriya and Faghih, 2020a):


(1) $$\begin{array}{ll} x_{j} & =x_{j-1}+\epsilon_{j}, \end{array}$$

where $\epsilon_{j}\sim N\left(0,\sigma_{\epsilon }^{2}\right)$ is the process noise and *j* stands for the time index. Following the marked point process filtering approach (Wickramasuriya and Faghih, 2020a), we consider Bernoulli distribution for neural impulse occurrence (arousal events) *n*_*j*_ with probability mass function $a_{j}^{n_{j}}{\left(1-a_{j}\right)}^{1-n_{j}}$ such that *P*(*n*_*j*_ = 1) = *a*_*j*_.

We can relate *x*_*j*_ to *a*_*j*_ by applying a sigmoid transform (Young et al., 2004). Thus,


(2) $$\begin{array}{ll} a_{j} & =\frac{1}{1+e^{-\left(x_{j}+\beta \right)}}, \end{array}$$

where β is a constant that can be derived from $\beta \approx \log\;\frac{a_{0}}{1-a_{0}}\;$, and *a*_0_ is the average probability of observing an impulse during the experiment. As described in Wickramasuriya and Faghih (2020a), the continuous-valued amplitude *r*_*j*_ of each neural impulse can be represented as


(3) $$\begin{array}{ll} r_{j} & =\gamma_{0}+\gamma_{1}x_{j}+v_{j}, \end{array}$$

where $v_{j}\sim N\left(0,\sigma_{v}^{2}\right)$ presents the sensor noise, γ_0_ and γ_1_ are the unknown model parameters in arousal state model in Equations (1)–(4), to be determined. Consequently, the joint density function for the observed neural impulse is


(4) $$\begin{array}{l} (n_{j}\cap r_{j}|x_{j})=\{\begin{array}{ll} 1-a_{j}\; & \text{if }n_{j}\text{=0}\\ a_{j}\frac{1}{\sqrt{2\pi \sigma_{v}^{2}}}e^{\frac{-{\left(r_{j}-\gamma_{0}-\gamma_{1}x_{j}\right)}^{2}}{2\sigma_{v}^{2}}} & \text{if }n_{j}\text{=1} \end{array}. \end{array}$$

The unknown parameters $\theta_{A}=\left\{\sigma_{\epsilon }^{2},\gamma_{0},\gamma_{1},\sigma_{v}^{2}\right\}$, and hidden arousal state *x*_*j*_ can be decoded at the same time using an expectation-maximization (EM) framework (Wickramasuriya and Faghih, 2020a). A description of the applied arousal state decoder is available in the Supplementary material.

#### 2.3.2 Performance state estimation

Inspired by the proposed state-space model in Prerau et al. (2009), we model the cognitive performance state as


(5) $$\begin{array}{l} z_{k}=z_{k-1}+w_{k}, \end{array}$$

where *z*_*k*_ is the performance state, $w_{k}\sim N\left(0,\sigma_{w}^{2}\right)$ stands for the process noise and *k* is the trial number during the experiment.

Similar to Prerau et al. (2009), we can form the observation model by specifying one binary observation (correct/incorrect response at *k*^*th*^ trial) and one continuous observation (reaction time of the corresponding response). The Bernoulli probability model is assumed for the binary responses with the probability mass function of $p_{k}^{m_{k}}{\left(1-p_{k}\right)}^{1-m_{k}}$. Applying sigmoid transform we express the *p*_*k*_ in terms of *z*_*k*_ such that


(6) $$\begin{array}{ll} p_{k} & =\frac{1}{1+e^{-\left(z_{k}+\mu \right)}}. \end{array}$$

The constant term μ can be derived from $\mu \approx \log\;\frac{p_{0}}{1-p_{0}}\;$ where *p*_0_ is the average probability of having a correct response.

The reaction time *t*_*k*_ can be related to the performance state as


(7) $$\begin{array}{ll} I_{k} & =\log t_{k}=\alpha_{0}+\alpha_{1}z_{k}+\delta_{k}, \end{array}$$

where we consider the log of reaction time at each trial (*I*_*k*_) to follow the linear model with the Gaussian noise term $\delta_{k}\sim N\left(0,\sigma_{\delta }^{2}\right)$; the vector of unknown model parameters in performance state model in Equations (5)–(7), $\theta_{P}=\left\{\sigma_{w}^{2},\alpha_{0},\alpha_{1},\sigma_{\delta }^{2}\right\}$ and the performance state *z*_*k*_ can be decoded using the EM approach (Prerau et al., 2009; Khazaei et al., 2021). A description of the applied performance state decoder is available in the Supplementary material.

#### 2.3.3 fNIRS processing

Similar to Yaghmour et al. (2021) and Parshi et al. (2019), we preprocess the collected hemodynamic data using the Nirslab software (Xu et al., 2014). The preprocessing steps include bandpass filtering and converting the light intensity data to HbO, HbR, and HbT concentrations (Yaghmour et al., 2021).

**Figure 4:**
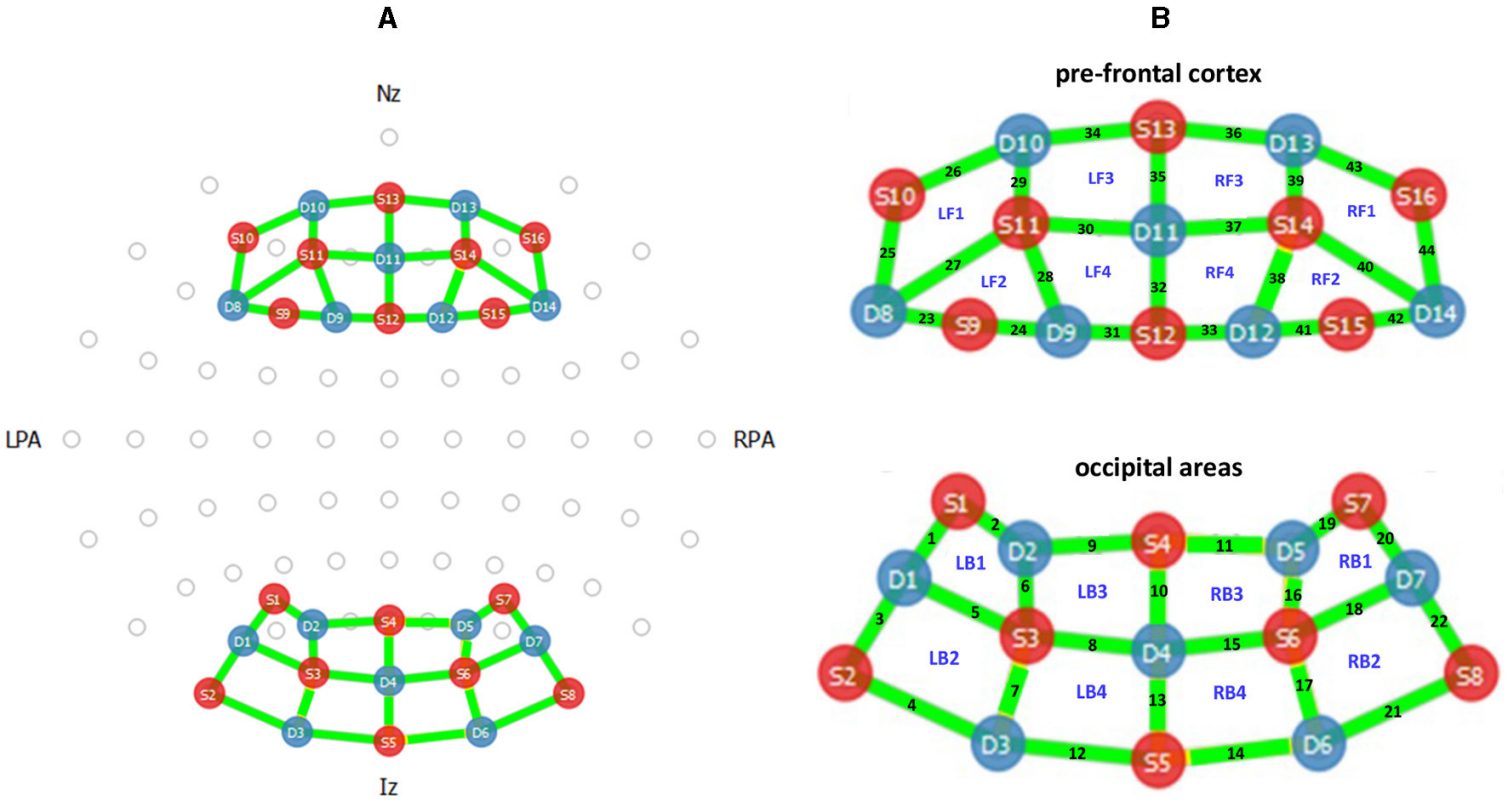
The Functional Near Infrared Spectroscopy (fNIRS) sensor configuration. **(A)** Optode layout of the fNIRS sources (red), detectors (blue), and channels (green) used during the experiment. The Nasion (Nz, the intersection point of the frontal and nasal bones), Inion (Iz, the occipital protuberance behind the scalp), and Left and Right Pre-auricular points (LPA, RPA, the points anterior to the ears in front of the upper end of the tragus) are labeled accordingly. **(B)** The channel numbers and studied regions of PFC and OC areas located on right and left hemisphere: LF1- to LF4, RF1 to RF4, LB1 to LB4, and RB1 to RB4.

We first analyze the HbO data. To study the PFC and OC areas, the combinations of HbO channels are considered such that we cover 16 different brain regions. These brain regions are distributed within the right and left sides of the PFC and OC areas (Donadel et al., 2021). As depicted in Figure 4B, each of the studied areas is surrounded by four channels, and the average HbO concentration can be derived accordingly. We evaluate the epoch of HbO signal over 22 trials (within each task block) of the *n*-back task with respect to the task difficulty (e.g., 1-back) and music session. The epoch of a signal can be defined as a signal segment within a specific time window. Such evaluation is inspired by the event-related potential (ERP) study, which analyzes a brainwave in response to the stimuli (Luck, 2014). Here, we consider the signal segment over 22 trials (≈44 s), and we perform a similar ERP-like study to derive an epoch of performance state with respect to the type of task (i.e., task difficulty) and music session. Then, we find the Pearson correlation coefficient between the epoch of HbO and continuous performance.

Additionally, inspired by the study in Wickramasuriya et al. (2023), we investigate HbT signal energy with respect to the presented music types. Particularly, we consider the collected signal from the PFC channels (i.e., channels 23–44) and smooth the signal using a 10 *s* sample-by-sample running average filter.

#### 2.3.4 Statistical analysis

The differences in human brain structure can lead to variation in behaviors, cognitive abilities, and mental and physical health (Gu and Kanai, 2014). To have a general and person-specific index of cognitive states with respect to an individual's baseline, we formulate the metrics called high arousal index (HAI) and high performance index (HPI) (Wickramasuriya and Faghih, 2020a). These arousal and performance indices can be calculated from *prob*(state_*j*_>threshold) where the threshold has been set to the median of the state values. Here, we decode the hidden arousal and performance state merely based on the SC and behavioral data (Fekri Azgomi et al., 2023). The HAI and HPI are evaluated with respect to task difficulty and music sessions. In particular, we perform the two-sided sign test to compare the 1-back task and 3-back task data (i.e., 1-back vs. 3-back) during each music session (*N* = 176); similarly, the two-sided sign test is executed to compare the HAI and HPI associated with calming and exciting sessions (calming vs. exciting) during each task difficulty level. We consider 0.01 as the significance level (99% confidence), and the *p*−values are reported in Tables 1, 2.

**Table 1:** The performed signrank test with respect to music sessions and *n*-back difficulty levels given the decoded high arousal index (HAI).

***p*** **-values**					
**Groups**	**Participant 1**	**Participant 2**	**Participant 3**	**Participant 4**	**Participant 5**
Calming session trials (1-back vs. 3-back)	0.0595	0.2582	1	0.3271	0.0129
Exciting session trials (1-back vs. 3-back)	0.821	0.0288	1.7349e-05	0.0012	0.5977
1-back trials (calming vs. exciting)	8.4957e-14	5.1376e-30	2.6388e-13	2.3808e-12	9.8706e-40
3-back trials (calming vs. exciting)	4.3804e-18	2.3884e-22	9.8706e-40	2.2015e-26	9.8706e-40

**Table 2:** The performed signrank test with respect to music sessions and *n*-back difficulty levels given the decoded high performance index (HPI).

***p*** **-values**					
**Groups**	**Participant 1**	**Participant 2**	**Participant 3**	**Participant 4**	**Participant 5**
Calming session trials (1-back vs. 3-back)	1.0693e-34	4.2545e-33	8.2346e-15	8.3044e-28	2.4552e-36
Exciting session trials (1-back vs. 3-back)	5.3702e-23	4.2647e-06	8.2346e-15	4.2545e-33	3.7427e-08
1-back trials (calming vs. exciting)	3.3866e-05	0.0033	4.4108e-07	3.9597e-04	0.8211
3-back trials (calming vs. exciting)	2.3808e-12	1.6276e-17	0.0053	2.1008e-16	1.1527e-18

## 3 Results

**Figure 5:**
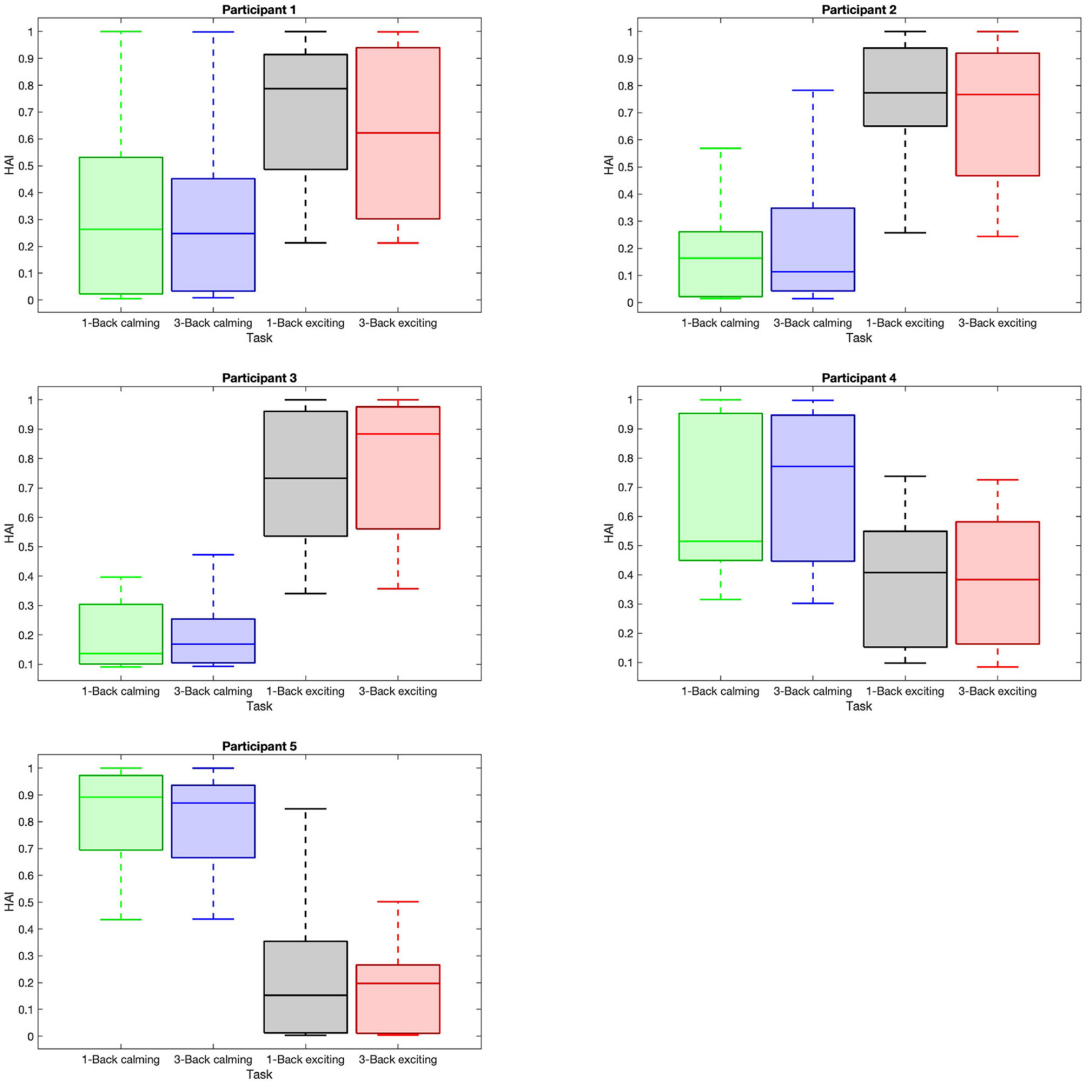
Distribution of high arousal index (HAI) within trials with respect to task difficulties and music sessions. Each sub-plot shows the box plot of the average HAI data within the trials with respect to 1-back task in the calming session (green box), 3-back task in the calming session (blue box), 1-back task in the exciting session (gray box), and 3-back task in the exciting session (red box).

Given the HAI boxplots in Figure 5, a higher variation in the arousal matrices with respect to the calming and exciting session is noted, while such variation can not be observed with respect to the task difficulty. The median values of HAI in 1-back and 3-back (1-back vs. 3-back) tasks do not diverge considerably. However, considering the music sessions (calming vs. exciting), the median values associated with the calming sessions do not fall within the range of exciting session boxes. According to the reported *p*-values and boxplots (Table 1), there is no significant difference between the HAI in calming session 1-back tasks and 3-back ones. In regards to HAI associated with exciting sessions, participants 3 and 4 are the only ones who depict a significant difference between the 1-back task and 3-back task data (Table 1). Considering the HAI with respect to the music, all the participants present significant differences when shifting from a calming to an exciting session (Table 1).

**Figure 6:**
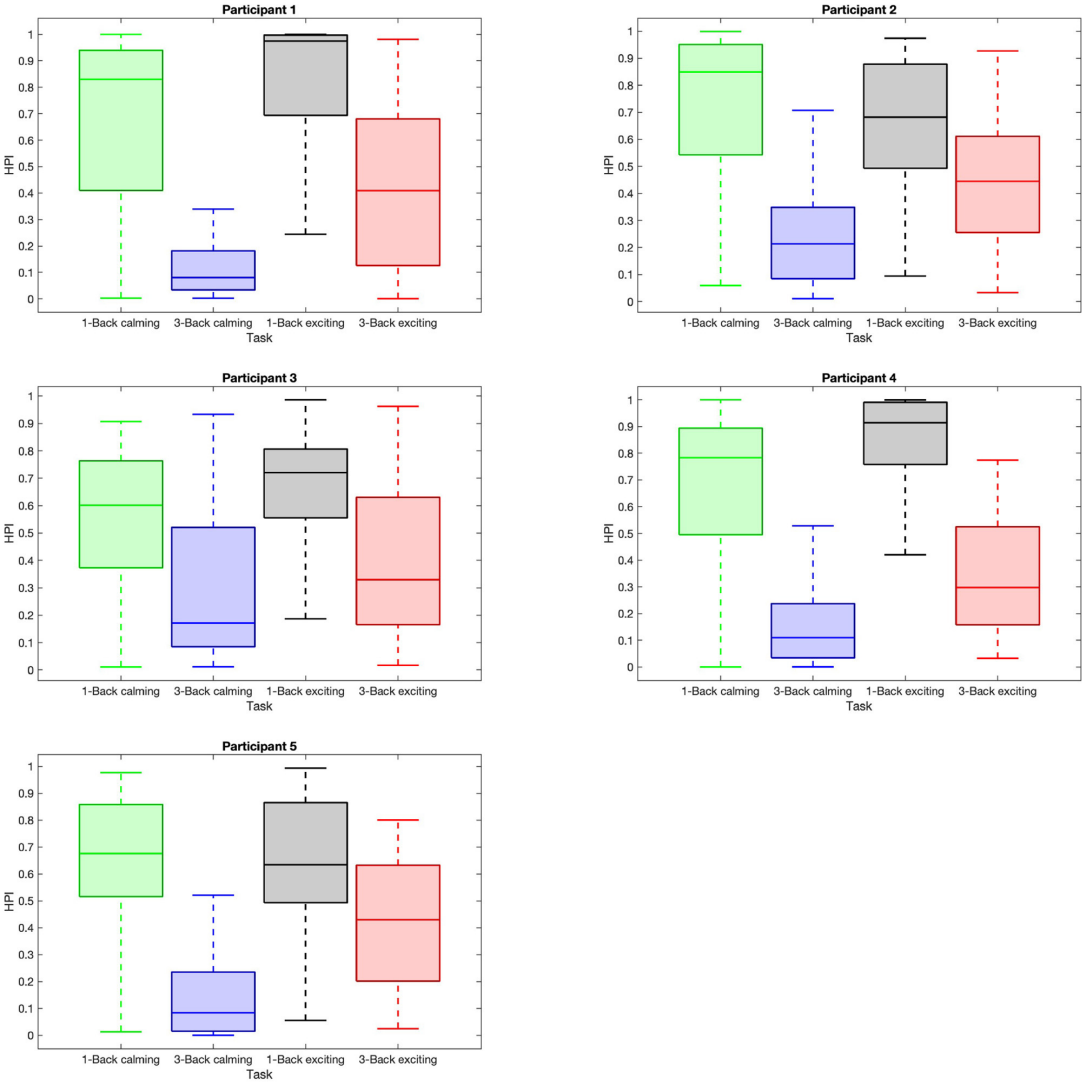
Distribution of high performance index (HPI) within trials with respect to task difficulties and music sessions. Each sub-plot shows the box plot of the HPI data within the trials with respect to 1-back task in the calming session (green box), 3-back task in the calming session (blue box), 1-back task in the exciting session (gray box), and 3-back task in the exciting session (red box).

We evaluate the HPI in a similar manner, and as expected, we find that the median of HPI within the 1-back task trials is considerably higher than in 3-back task trials (Figure 6). Also, the HPI median values among all the participants are higher within the 3-back task trials during the exciting session compared to the calming session (Figure 6). The reported *p*−values in Table 2 depict the significant difference in HPI with respect to the task difficulty as well as music session in most of the cases.

**Figure 7:**
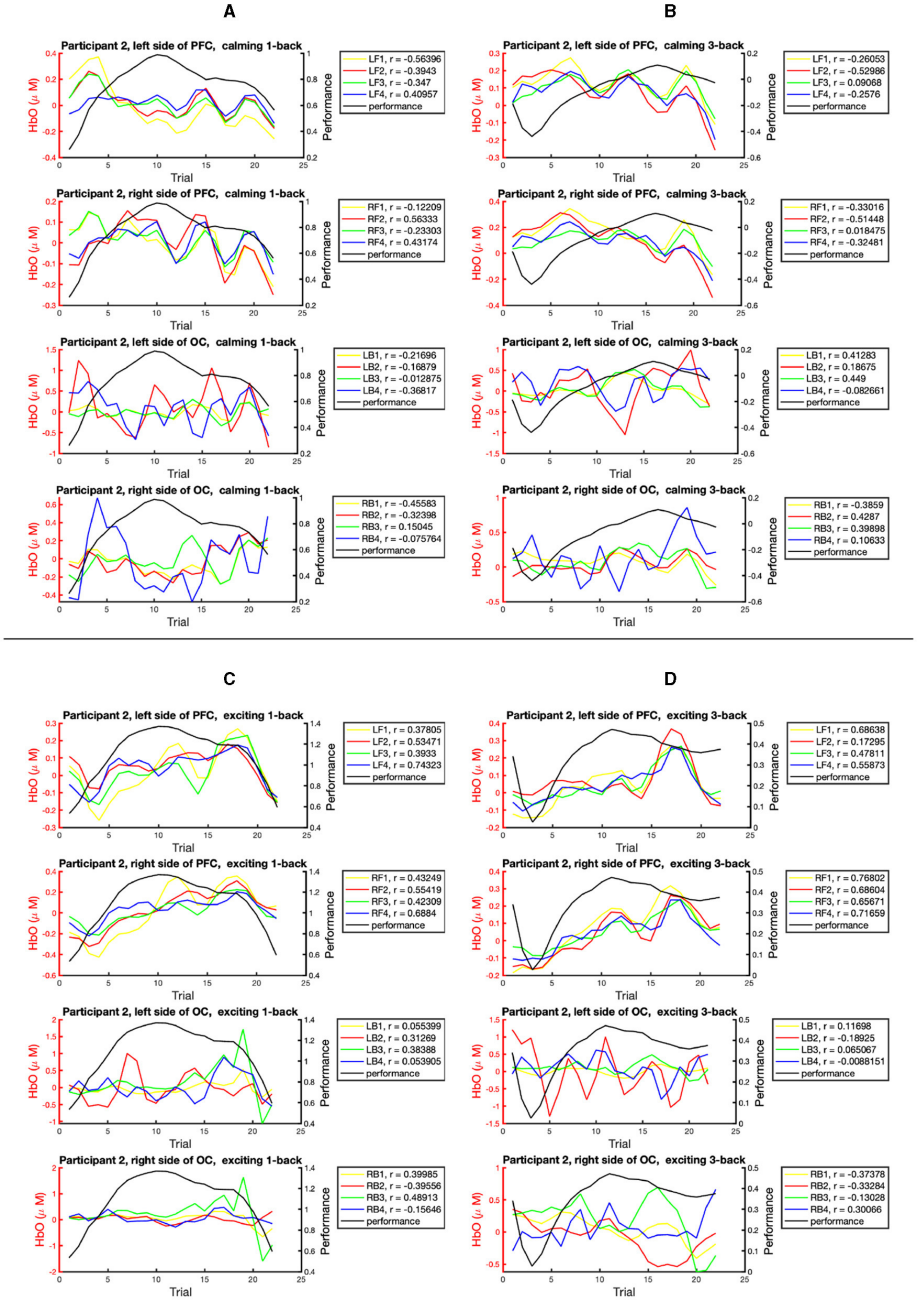
The correlation study for the epoch of HbO and performance state across the task blocks for one participant. The sub-figures present the epochs of HbO and performance data recorded within: **(A)** the 1-back task blocks within the calming music. **(B)** the 3-back task blocks within the calming music. **(C)** the 1-back task blocks within the exciting music. **(D)** the 3-back task blocks within the exciting music. The sub-plots in each sub-figure, from top to bottom, represent: the HbO data collected from the left side of the PFC, right side of the PFC, left side of the OC area, and right side of the OC area. The boxes on the right side of subplots present the Pearson correlation coefficients (*r*) within the studied brain regions.

Figure 7 presents the epochs of HbO concentration and performance across the task blocks for participant 2. As noted earlier, 16 specific brain regions from the left and right sides of the PFC and OC areas are considered. The correlation between the HbO epochs and performance epochs with respect to the types of the *n*-back task and the played music is illustrated. In particular, Figure 7A displays the correlation in the 1-back task blocks within the calming session; Figure 7B pays attention to the 3-back task blocks within the calming session; Figure 7C addresses the 1-back task blocks within the exciting session; Figure 7D considers the 3-back task blocks within the exciting session. The Pearson correlation coefficient (*r*) of performance and HbO concentration for each studied brain region is reported in a box next to each subplot. The highest HbO and performance positive correlation for participant 2 corresponds to the RF1 region (right side of the PFC) during the 3-back task blocks within the exciting session. Similar demonstrations for the other participants can be found in the Supplementary material. Also, the highest positive correlation can be observed within the 3-back task blocks for all participants. In particular, we can see that the highest correlation in participants 1–5 corresponds to LB1, RF1, LF4, LF3, and LF2, respectively. Four out of five participants presented the highest performance-HbO correlation with the acquired HbO signal from the left hemisphere. A similar trend can be noted concerning the PFC areas.

**Figure 8:**
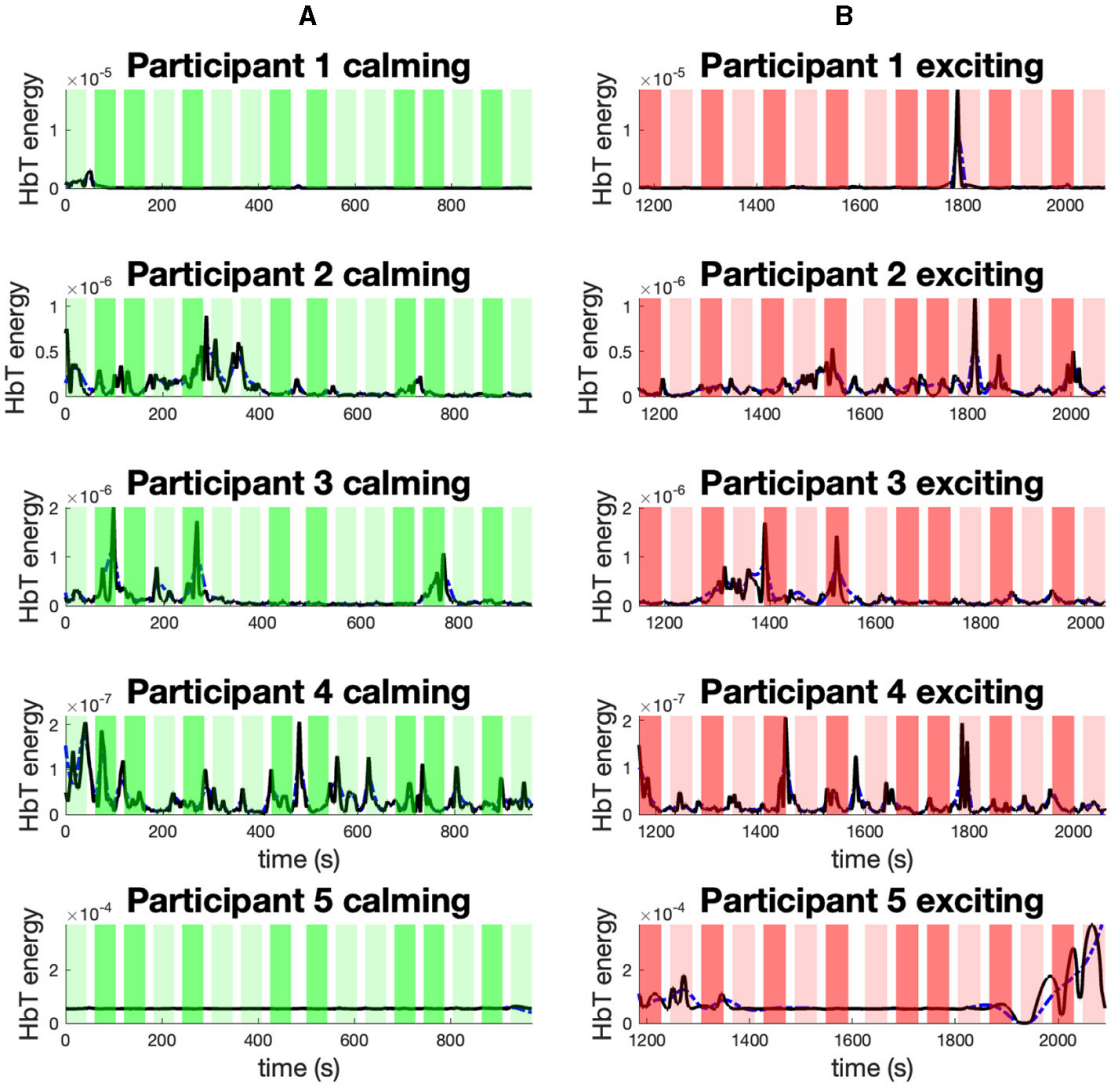
The mean energy and mean envelope of smoothed HbT signal collected from PFC channels. The sub-figures present: **(A)** The mean energy (black) and mean envelope (blue) of smoothed HbT signal collected from PFC channels within the calming music for participants 1–5. **(B)** The mean energy (black) and mean envelope (blue) of smoothed HbT signal collected from PFC channels within the exciting music for participants 1–5. The background colors in each sub-panel indicate: the 1-back task during the calming session (light green); the 3-back task during the calming session (dark green); the 1-back task during the exciting session (light red); the 3-back task during the exciting session (dark red).

In Figure 8, we present the mean energy (black) and mean envelope (blue) of smoothed HbT signal collected from the PFC channels (i.e., channels 23–44). The Figure 8A presents the results within the calming session (green), and Figure 8B is related to the exciting session (red). The 1-back task blocks are indicated with lighter colors, while the 3-back ones are represented with more intense background colors. Aside from participant 3, it can be seen that the peak of the HbT energy is located within the exciting session.

## 4 Discussion

We have performed a working memory experiment in the presence of musical stimuli to collect multimodal physiological signals along with brain hemodynamic response signals of participants and evaluate the possibility of using music as an intervention. Participants were asked to perform a working memory task with different difficulty levels and music types (i.e., calming and exciting). The difficulty levels were included to ensure different cognitive loads during the experiment. We have used fNIRS to record the brain hemodynamic response. By incorporating multiple physiological signals in the experimental data collection, we have derived a rich neuro-physiological dataset that could offer a more comprehensive picture of the body and brain's reaction to music and cognitive load. In this dataset, we have implemented the musical stimuli by conducting the cognitive task in two sessions–one with calming music content and the other with exciting music content. The presence of calming music was supposed to mimic the low arousal condition, while the exciting one was simulating the high arousal one. To incorporate the personalized version of CLBMI, we have used personalized music selected by the participants for calming and exciting music. While the applied music intervention offers us a personalized closed-loop architecture, it can induce the subject familiarity with the experiment environment. One possible approach to preserve the person-specific nature of the intervention and reduce the impact of the subject's familiarity is to employ newly generated music based on the subject's preference in future studies (Fekri Azgomi et al., 2023).

In the presented research, we have collected data using both research grade (e.g., Biopac) and wearable devices (e.g., Empatica E4). While wearable devices can be implemented in everyday life settings and they seem to be more aligned with the future closed-loop architecture, the employed signal processing algorithms and estimation framework are more compatible with the research grade devices, and they outperform in the lab settings. Hence, we perform our in-depth analysis based on the data collected using research-grade devices. Although the presented framework includes a multimodal measurement that can be used concurrently for brain state estimation and classification, here, we analyze the SC data, oxygenated hemoglobin, total hemoglobin, and behavioral signals. However, we deliver complete dataset and experimental settings to provide a framework for any researcher who is interested in performing research in this paradigm. Particularly, the collected data presents a unique opportunity for future investigations, and it holds the potential to unlock groundbreaking insights and guide future data collection in this context.

Recording multimodal physiological signals within a cognitive task can provide an opportunity to explore cutting-edge strategies that may extend human cognitive capacities (Mangaroska et al., 2021). However, multimodal physiological data collection comes with certain challenges (Cukurova et al., 2020). Specifically, a suboptimal sensor connection or the presence of motion artifact in the modalities can lead to data corruption (Mangaroska et al., 2021; Fekri Azgomi et al., 2023). Consequently, the participant removal rate in this form of research, as observed in this experiment, can be high. Given the fast-paced advances in biomedical sensor development, one potential approach to address this concern is to use a more advanced setup that employs a lower number of sensors with a more robust connection to collect the signal of interest. Employing the recently developed sensors may pave the way for future data collection in a more practical and efficient manner.

Based on our analysis on the SC signal, we can observe that the HAI level varies significantly within the music sessions, while the task-wise perspective does not reveal a significant variation. One may interpret that the difference in the induced cognitive load by 1-back and 3-back tasks is not significantly high enough to be reflected in the HAI signal. Another interpretation, though less probable, is that the impact of cognitive load on the arousal level is not as much as the effect of music type, and the cognitive load does not seem to have a mediating effect on arousal. Determining a general relationship that holds out of the scope of this dataset requires a comprehensive causality analysis, which is not the main focus of this study.

It should be noted that the HAI for participants 4 and 5 was reduced within the exciting session. This is the opposite of what the exciting content of music is supposed to elicit. This is a notable counterexample and demonstrates that the skin conductance response does not agree with the emotional content of music for participants 4 and 5. On the other hand, we can see that the HbT energy signal reaches its peak within the exciting session in both participant 4 and 5 cases. While the SC is a long-standing index of arousal (Greco et al., 2016), we can observe that decoding the arousal from a single SC measurement might lack the robustness needed to explain the observed behavior. To address this concern in future investigations and experiment designs, the graphene e-tattoos (GET) sensors can be applied (Jang et al., 2022), and the SC data can be collected and analyzed in a multichannel manner (Alam et al., 2023).

According to the presented HPI, except for participant 2, we can see that the performance indices are elevated within the second session, in which the exciting music was presented. While one may hypothesize that the improved performance is a result of establishing the arousal within a moderate range using the exciting music, other factors such as the learning impact and participant's familiarity with the environment can be influential, and we should avoid making any definite conclusion on the impact of music. Here, findings may suggest the potential for integrating music into the closed-loop system. Given the low sample size, the absence of any mental state score, and the possible confounding factors, further studies with a higher number of subjects, mental state annotation, inclusion of a control group, and shuffled task difficulty as well as music sessions are required for a decisive resolution.

In the context of BMI, users may experience various cognitive loads and emotional states during the interaction with a technical system (Herff et al., 2014). Both cognitive load and emotion status can trigger a particular brain response followed by variation in the cognitive performance (Fishburn et al., 2014; Bigliassi et al., 2015). Hence, it is crucial to understand the relation between the brain response under different cognitive loads and environmental stimuli to potentially optimize performance. The performed experiment provides an opportunity to study the hemodynamic response within different cognitive loads and music sessions. The highest HbO and performance positive correlation for participants 1–5 can be seen during the 3-back task blocks (Supplementary material). One may interpret that the HbO data has the potential to be applied as an informative biomarker of performance within high cognitive loads. Also, the observed high HbT energy over the PFC within exciting sessions may be interpreted as participants' higher brain activities within the exciting session compared to the calming session. This conforms with the findings in Zheng et al. (2020). Perhaps the participants had to concentrate more within higher arousal levels (Wickramasuriya et al., 2023). Overall, these findings may be an indication of a new avenue for decoder design research, leading to innovative fNIRS feature extraction to decode the hidden arousal and performance states. It is important to highlight that due to the small sample size, further studies with a higher number of participants and more interventions as well as cognitive loads would be beneficial in drawing a final conclusion.

It would be vital to note that the experiment serves as a pilot study to investigate the viability of using music as the brain state regulator. In general, our findings show variation in collected signals as well as decoded brain states between different music sessions. According to each person's unique physiology and the applied personalized intervention, we are interested in having a personalized perspective rather than a general view. Particularly, the brain structure of individuals and the network-level interaction between cognitive brain states of individuals can be studied independently regardless of the sample size (Hurlburt et al., 2015). The preliminary findings show that music may potentially have ramifications in the BMI system as a form of background stimulation, while more in-depth research is required to fully understand the role of music on arousal and performance. Specifically, in the CLBMI realm, it is crucial to have precise and robust measurements as well as feedback mechanisms. The presented pilot study should be improved to ensure the robustness of the CLBMI pathways. In the future, magnetoencephalography (MEG) can be employed in parallel with fNIRS technique to improve the spatial and temporal resolution of the brain recordings and implement the neurofeedback in our setup (Yucha and Montgomery, 2008). We intend this study to be a stepping stone for formulating such studies and developing more comprehensive experiments.

## Data availability statement

The raw data supporting the conclusions of this article will be made available by the authors, without undue reservation.

## Ethics statement

The studies involving humans were approved by Institutional Review Board at the University of Houston, TX, USA. The studies were conducted in accordance with the local legislation and institutional requirements. The participants provided their written informed consent to participate in this study.

## Author contributions

SK: Data curation, Investigation, Methodology, Software, Visualization, Writing – original draft, Writing – review & editing, Formal analysis, Validation. SP: Investigation, Methodology, Visualization, Writing – original draft, Writing – review & editing. SA: Data curation, Writing – original draft, Writing – review & editing. MA: Data curation, Investigation, Methodology, Writing – review & editing. RF: Conceptualization, Funding acquisition, Investigation, Methodology, Resources, Supervision, Writing – review & editing.

## References

[B1] Alam S. Amin M. R. Faghih R. T. (2023). Sparse multichannel decomposition of electrodermal activity with physiological priors. IEEE Open J. Eng. Med. Biol. 4, 234–250. 10.1109/OJEMB.2023.3332839 38196978 PMC10776104

[B2] Amin M. R. Tahir M. Faghih R. T. (2021). “A state-space investigation of impact of music on cognitive performance during a working memory experiment,” in 2021 43rd Annual International Conference of the IEEE Engineering in Medicine and Biology Society (EMBC) (Mexico: IEEE), 757–762. 10.1109/EMBC46164.2021.9629632 34891401

[B3] Amin R. Faghih R. T. (2022). Physiological characterization of electrodermal activity enables scalable near real-time autonomic nervous system activation inference. PLoS Comput. Biol. 18:e1010275. 10.1371/journal.pcbi.1010275 35900988 PMC9333288

[B4] Ayaz H. Izzetoglu M. Bunce S. Heiman-Patterson T. Onaral B. (2007). “Detecting cognitive activity related hemodynamic signal for brain computer interface using functional near infrared spectroscopy,” in 2007 3rd International IEEE/EMBS Conference on Neural Engineering (Kohala Coast, HI: IEEE), 342–345.

[B5] Baddeley A. (1992). Working memory. Science 255, 556–559. 1736359 10.1126/science.1736359

[B6] Baldwin C. L. Penaranda B. (2012). Adaptive training using an artificial neural network and EEG metrics for within- and cross-task workload classification. NeuroImage 59, 48–56. 10.1016/j.neuroimage.2011.07.047 21835243

[B7] Basu I. Yousefi A. Crocker B. Zelmann R. Paulk A. C. Peled N.. (2023). Closed-loop enhancement and neural decoding of cognitive control in humans. Nat. Biomed. Eng. 7, 576–588. 10.1038/s41551-021-00804-y 34725508 PMC9056584

[B8] Berka C. Levendowski D. J. Lumicao M. N. Yau A. Davis G. Zivkovic V. T.. (2007). EEG correlates of task engagement and mental workload in vigilance, learning, and memory tasks. Aviat. Space Environ. Med. 78(5Suppl.), 231–244. Available online at: https://www.ingentaconnect.com/content/asma/asem/2007/00000078/a00105s1/art00032 17547324

[B9] Bigliassi M. Barreto-Silva V. Altimari L. R. Vandoni M. Codrons E. Buzzachera C. F. (2015). How motivational and calm music may affect the prefrontal cortex area and emotional responses: a functional near-infrared spectroscopy (fnirs) study. Percept. Mot. Skil. 120, 202–218. 10.2466/27.24.PMS.120v12x5 25650505

[B10] Cain B. (2007). A Review of the Mental Workload Literature. Technical Report. Toronto, ON: Defence Research and Development.

[B11] Cudo A. Francuz P. Augustynowicz P. Stróżak P. (2018). The effects of arousal and approach motivated positive affect on cognitive control. An ERP study. Front. Hum. Neurosci. 12:320. 10.3389/fnhum.2018.00320 30233339 PMC6128242

[B12] Cukurova M. Giannakos M. Martinez-Maldonado R. (2020). The promise and challenges of multimodal learning analytics. Br. J. Educ. Technol. 51, 1441–1449. 10.1111/bjet.13015

[B13] Donadel D. G. Zortea M. Torres I. L. Fregni F. Caumo W. (2021). The mapping of cortical activation by near-infrared spectroscopy might be a biomarker related to the severity of fibromyalgia symptoms. Sci. Rep. 11:15754. 10.1038/s41598-021-94456-2 34344913 PMC8333354

[B14] Ehrlich S. K. Agres K. R. Guan C. Cheng G. (2019). A closed-loop, music-based brain-computer interface for emotion mediation. PLoS ONE 14:e0213516. 30883569 10.1371/journal.pone.0213516 PMC6422328

[B15] Fazli S. Mehnert J. Steinbrink J. Curio G. Villringer A. Müller K. R.. (2012). Enhanced performance by a hybrid NIRS—EEG brain computer interface. NeuroImage 59, 519–529. 10.1016/j.neuroimage.2011.07.084 21840399

[B16] Fekri Azgomi H. F. Branco L. R. Amin M. R. Khazaei S. Faghih R. T. (2023). Regulation of brain cognitive states through auditory, gustatory, and olfactory stimulation with wearable monitoring. Sci. Rep. 13:12399. 10.1038/s41598-023-37829-z 37553409 PMC10409795

[B17] Fishburn F. A. Norr M. E. Medvedev A. V. Vaidya C. J. (2014). Sensitivity of fnirs to cognitive state and load. Front. Hum. Neurosci. 8:76. 10.3389/fnhum.2014.00076 24600374 PMC3930096

[B18] Fyshe A. Fox E. Dunson D. Mitchell T. (2012). “Hierarchical latent dictionaries for models of brain activation,” in Proceedings of the Fifteenth International Conference on Artificial Intelligence and Statistics, Vol. 22 of Proceedings of Machine Learning Research, eds. N. D. Lawrence and M. Girolami (La Palma: PMLR), 409–421.

[B19] Greco A. Valenza G. Scilingo E. P. (2016). Advances in Electrodermal Activity Processing With Applications for Mental Health. Berlin: Springer.

[B20] Gu J. Kanai R. (2014). What contributes to individual differences in brain structure? Front. Hum. Neurosci. 8:262. 10.3389/fnhum.2014.00262 24808848 PMC4009419

[B21] Herff C. Heger D. Fortmann O. Hennrich J. Putze F. Schultz T. (2014). Mental workload during n-back task-quantified in the prefrontal cortex using fNIRS. Front. Hum. Neurosci. 7:935. 10.3389/fnhum.2013.00935 24474913 PMC3893598

[B22] Hobson J. A. Lindsley D. B. (1988). Activation, arousal, alertness, and attention. Stat. Brain Mind 1988, 1–3.

[B23] Hoshi Y. Tsou B. H. Billock V. A. Tanosaki M. Iguchi Y. Shimada M.. (2003). Spatiotemporal characteristics of hemodynamic changes in the human lateral prefrontal cortex during working memory tasks. Neuroimage 20, 1493–1504. 10.1016/S1053-8119(03)00412-9 14642462

[B24] Huang R. H. Shih Y. N. (2011). Effects of background music on concentration of workers. Work 38, 383–387. 10.3233/WOR-2011-1141 21508527

[B25] Hurlburt R. T. Alderson-Day B. Fernyhough C. Kühn S. (2015). What goes on in the resting-state? A qualitative glimpse into resting-state experience in the scanner. Front. Psychol. 6:1535. 10.3389/fpsyg.2015.01535 26500590 PMC4597037

[B26] Jang H. Sel K. Kim E. Kim S. Yang X. Kang S.. (2022). Graphene e-tattoos for unobstructive ambulatory electrodermal activity sensing on the palm enabled by heterogeneous serpentine ribbons. Nat. Commun. 13:6604. 10.1038/s41467-022-34406-2 36329038 PMC9633646

[B27] Khazaei S. Amin M. R. Faghih R. T. (2021). “Decoding a neurofeedback-modulated cognitive arousal state to investigateperformance regulation by the Yerkes-Dodson law,” in 2021 43rd Annual International Conference of the IEEE Engineering in Medicine and Biology Society (EMBC) (Mexico: IEEE). 10.1109/EMBC46164.2021.9629764 34892610

[B28] Khazaei S. Amin R. Tahir M. Faghih R. T. (2024). Bayesian inference of hidden cognitive performance and arousal states in presence of music. IEEE Open J. Eng. Med. Biol. 2024:3377923. 10.1109/OJEMB.2024.3377923 PMC11342937 39184959

[B29] Kuschpel M. S. Liu S. Schad D. J. Heinzel S. Heinz A. Rapp M. A. (2015). Differential effects of wakeful rest, music and video game playing on working memory performance in the N-back task. Front. Psychol. 6:1683. 10.3389/fpsyg.2015.01683 26579055 PMC4626555

[B30] Luck S. J. (2014). An Introduction to the Event-Related Potential Technique. Cambridge, MA: MIT Press.

[B31] Mangaroska K. Martinez-Maldonado R. Vesin B. Gašević D. (2021). Challenges and opportunities of multimodal data in human learning: The computer science students' perspective. J. Comput. Assist. Learn. 37, 1030–1047. 10.1111/jcal.1254227409075

[B32] McGaugh J. L. (2004). The amygdala modulates the consolidation of memories of emotionally arousing experiences. Annu. Rev. Neurosci. 27, 1–28. 10.1146/annurev.neuro.27.070203.144157 15217324

[B33] Meidenbauer K. L. Choe K. W. Cardenas-Iniguez C. Huppert T. J. Berman M. G. (2021). Load-dependent relationships between frontal fnirs activity and performance: a data-driven PLS approach. NeuroImage 230:117795. 10.1016/j.neuroimage.2021.117795 33503483 PMC8145788

[B34] Oostenveld R. Praamstra P. (2001). The five percent electrode system for high-resolution EEG and ERP measurements. Clin. Neurophysiol. 112, 713–719. 10.1016/S1388-2457(00)00527-7 11275545

[B35] Ottonello M. Fiabane E. Pistarini C. Spigno P. Torselli E. (2019). Difficulties in emotion regulation during rehabilitation for alcohol addiction: correlations with metacognitive beliefs about alcohol use and relapse risk. Neuropsychiat. Dis. Treat. 2019, 2917–2925. 10.2147/NDT.S214268 31686826 PMC6798816

[B36] Parshi S. Amin R. Azgomi H. F. Faghih R. T. (2019). “Mental workload classification via hierarchical latent dictionary learning: a functional near infrared spectroscopy study,” in 2019 IEEE EMBS International Conference on Biomedical and Health Informatics (BHI) (Chicago, IL: IEEE), 1–4.

[B37] Prerau M. J. Smith A. C. Eden U. T. Kubota Y. Yanike M. Suzuki W.. (2009). Characterizing learning by simultaneous analysis of continuous and binary measures of performance. J. Neurophysiol. 102, 3060–3072. 10.1152/jn.91251.2008 19692505 PMC2777819

[B38] Ray K. D. Mittelman M. S. (2017). Music therapy: a nonpharmacological approach to the care of agitation and depressive symptoms for nursing home residents with dementia. Dementia 16, 689–710. 10.1177/1471301215613779 26519453

[B39] Roy R. N. Bonnet S. Charbonnier S. Campagne A. (2013). “Mental fatigue and working memory load estimation: interaction and implications for EEG-based passive BCI,” in 2013 35th Annual International Conference of the IEEE Engineering in Medicine and Biology Society (EMBC) (Osaka: IEEE), 6607–6610. 10.1109/EMBC.2013.6611070 24111257

[B40] Salas C. E. Gross J. J. Turnbull O. H. (2019). Using the process model to understand emotion regulation changes after brain injury. Psychol. Neurosci. 12:430. 10.1037/pne0000174

[B41] Santosa H. Zhai X. Fishburn F. Huppert T. (2018). The nirs brain analyzir toolbox. Algorithms 11:73. 10.3390/a11050073 38957522 PMC11218834

[B42] Sassaroli A. Fantini S. (2004). Comment on the modified beer—lambert law for scattering media. Phys. Med. Biol. 49:N255. 10.1088/0031-9155/49/14/N07 15357206

[B43] Shin J. von Lühmann A. Kim D. W. Mehnert J. Hwang H. J. Müller K.-R. (2018). Simultaneous acquisition of EEG and NIRS during cognitive tasks for an open access dataset. Sci. Data 5:3. 10.1038/sdata.2018.3 29437166 PMC5810421

[B44] Struckmann W. Persson J. Gingnell M. Weigl W. Wass C. Bodén R. (2021). Unchanged cognitive performance and concurrent prefrontal blood oxygenation after accelerated intermittent theta-burst stimulation in depression: a sham-controlled study. Front. Psychiatr. 12:659571. 10.3389/fpsyt.2021.659571 34276437 PMC8278060

[B45] Ünal A. B. de Waard D. Epstude K. Steg L. (2013). Driving with music: effects on arousal and performance. Transport. Res. F Traf. Psychol. Behav. 21, 52–65. 10.1016/j.trf.2013.09.004

[B46] Ünal A. B. Steg L. Epstude K. (2012). The influence of music on mental effort and driving performance. Accid. Anal. Prev. 48, 271–278. 10.1016/j.aap.2012.01.022 22664690

[B47] Villringer A. Planck J. Hock C. Schleinkofer L. Dirnagl U. (1993). Near infrared spectroscopy (NIRS): a new tool to study hemodynamic changes during activation of brain function in human adults. Neurosci. Lett. 154, 101–104. 8361619 10.1016/0304-3940(93)90181-j

[B48] von Janczewski N. Wittmann J. Engeln A. Baumann M. Krauß L. (2021). A meta-analysis of the n-back task while driving and its effects on cognitive workload. Transport. Res. F. Traf. Psychol. Behav. 76, 269–285. 10.1016/j.trf.2020.11.014

[B49] Wendel K. Väisänen O. Malmivuo J. Gencer N. G. Vanrumste B. Durka P. (2009). EEG/MEG source imaging: methods, challenges, and open issues. Comput. Intell. Neurosci. 2009:13. 10.1155/2009/656092 19639045 PMC2715569

[B50] Wickramasuriya D. S. Amin M. Faghih R. T. (2019). Skin conductance as a viable alternative for closing the deep brain stimulation loop in neuropsychiatric disorders. Front. Neurosci. 13:780. 10.3389/fnins.2019.00780 31447627 PMC6692489

[B51] Wickramasuriya D. S. Crofford L. J. Widge A. S. Faghih R. T. (2022). Hybrid decoders for marked point process observations and external influences. IEEE Trans. Biomed. Eng. 2022:3191243. 10.1109/TBME.2022.3191243 35839187

[B52] Wickramasuriya D. S. Faghih R. T. (2019a). A Bayesian filtering approach for tracking arousal from binary and continuous skin conductance features. IEEE Trans. Biomed. Eng. 67, 1749–1760. 10.1109/TBME.2019.2945579 31603767

[B53] Wickramasuriya D. S. Faghih R. T. (2019b). “A novel filter for tracking real-world cognitive stress using multi-time-scale point process observations,” in 2019 41st Annual International Conference of the IEEE Engineering in Medicine and Biology Society (EMBC) (Berlin: IEEE), 599–602. 10.1109/EMBC.2019.8857917 31945969

[B54] Wickramasuriya D. S. Faghih R. T. (2020a). A marked point process filtering approach for tracking sympathetic arousal from skin conductance. IEEE Access 8, 68499–68513. 10.1109/ACCESS.2020.2984508

[B55] Wickramasuriya D. S. Faghih R. T. (2020b). A mixed filter algorithm for sympathetic arousal tracking from skin conductance and heart rate measurements in pavlovian fear conditioning. PLoS ONE 15:e0231659. 10.1371/journal.pone.0231659 32324756 PMC7179889

[B56] Wickramasuriya D. S. Khazaei S. Kiani R. Faghih R. T. (2023). A Bayesian filtering approach for tracking sympathetic arousal and cortisol-related energy from marked point process and continuous-valued observations. IEEE Access 11, 137204–137247. 10.1109/ACCESS.2023.3334974

[B57] Wickramasuriya D. S. Qi C. Faghih R. T. (2018). A state-space approach for detecting stress from electrodermal activity. Annu. Int. Conf. IEEE Eng. Med. Biol. Soc. 2018, 3562–3567. 10.1109/EMBC.2018.8512928 30441148

[B58] Xu Y. raber H. L. Barbour R. L. (2014). “nirsLAB: a computing environment for fNIRS neuroimaging data analysis,” in Biomedical Optics 2014 OSA Technical Digest (Online) (Miami, FL: Optical Society of America). 10.1364/BIOMED.2014.BM3A.1

[B59] Yaghmour A. Amin M. R. Faghih R. T. (2021). Decoding a music-modulated cognitive arousal state using electrodermal activity and functional near-infrared spectroscopy measurements. Annu. Int. Conf. IEEE Eng. Med. Biol. Soc. 2021, 1055–1060. 10.1109/EMBC46164.2021.9630879 34891470

[B60] Yerkes R. M. (1907). The Dancing Mouse, Vol. 1. New York, NY: Macmillan Company.

[B61] Yerkes R. M. Dodson J. D. (1908). The relation of strength of stimulus to rapidity of habit-formation. Punishment 1908, 27–41.

[B62] Young P. M. Clegg B. A. Smith C. A. P. (2004). Dynamic models of augmented cognition. Int. J. Hum. Comput. Interact. 17, 259–273. 10.1207/s15327590ijhc1702_833486653

[B63] Yucha C. Montgomery D. (2008). Evidence-Based Practice in Biofeedback and Neurofeedback. Ridge, CO: AAPB Wheat.

[B64] Zheng M. Lin H. Chen F. (2020). An fNIRS study on the effect of music style on cognitive activities. Annu. Int. Conf. IEEE Eng. Med. Biol. Soc. 2020, 3200–3203. 10.1109/EMBC44109.2020.9176441 33018685

