# Supplementary material for: A multimodal dataset for investigating working memory in presence of music: a pilot study
Source: Front Neurosci. 2024 Jun 19;18:1406814. doi: 10.3389/fnins.2024.1406814 (PMC11220373; doi:10.3389/fnins.2024.1406814)
Supplement: Supplementary file 1 [file Data_Sheet_1.pdf]

Supplementary Material

1 SUPPLEMENTARY METHODS

1.1 Arousal State Decoder

To decode the hidden arousal, we apply the expectation-maximization (EM) framework. This type of arousal decoder originally derived in (Wickramasuriya and Faghih, 2020).

The E-step equations can be formed according to observation $R^J = \{(n_1, r_1), ..., (n_J, r_J)\}$ up to time J. We apply Bayesian filtering to estimate x_j. The E-step includes the forward filter and backward smoother presented below.

Predict:

$$x_{j|j-1} = x_{j-1|j-1}, \tag{S1}$$

$$\sigma^2_{j|j-1} = \sigma^2_{j-1|j-1} + \sigma^2_{\epsilon}, \tag{S2}$$

Update:

if $n_j = 0$

$$x_{j|j} = x_{j|j-1} + \sigma^2_{j|j-1}(n_j - a_{j|j}), \tag{S3}$$

$$\sigma^2_{j|j} = \left[\frac{1}{\sigma^2_{j|j-1}} + a_{j|j}(1 - a_{j|j})\right]^{-1}, \tag{S4}$$

if $n_j = 1$

$$C_j = \frac{\sigma^2_{j|j-1}}{\gamma_1^2\sigma^2_{j|j-1} + \sigma^2_v}, \tag{S5}$$

$$x_{j|j} = x_{j|j-1} + C_j\Big[\sigma^2_v(n_j - a_{j|j}) + \gamma_1(r_j - \gamma_0 - \gamma_1 x_{j|j-1})\Big], \tag{S6}$$

$$\sigma^2_{j|j} = \left[\frac{1}{\sigma^2_{j|j-1}} + a_{j|j}(1 - a_{j|j}) + \frac{\gamma_1^2}{\sigma^2_v}\right]^{-1}. \tag{S7}$$

To implement a backward smoother, we reverse the direction and improve x_j by obtaining a set of smoothed mean and variance estimates:

$$A_j = \frac{\sigma^2_{j|j}}{\sigma^2_{j+1|j}}, \tag{S8}$$

$$x_{j|J} = x_{j|j} + A_j(x_{j+1|J} - x_{j+1|j}), \tag{S9}$$

$$\sigma^2_{j|J} = \sigma^2_{j|j} + A_j^2(\sigma^2_{j+1|J} - \sigma^2_{j+1|j}). \tag{S10}$$

In the M-step, we the apply E-step results and find the unknown parameters such that they maximize the log-likelihood function. To move to the M-step, we first calculate the expected values of x_j^2, and $x_j x_{j-1}$:

$$\mathbb{E}[x_j^2] = x^2_{j|J} + \sigma^2_{j|J}, \tag{S11}$$

$$\mathbb{E}[x_{j+1}x_j] = x_{j+1|J}x_{j|J} + A_j\sigma^2_{j+1|J}. \tag{S12}$$

We derive the log-likelihood function Q_1, and find the unknown parameters that maximize the expected value of Q_1;

$$\mathbb{E}[Q_1] = \sum_{j=1}^{J} \mathbb{E}[n_j(\beta + x_j) - \log(1 + e^{\beta + x_j})] \tag{S13}$$
$$+ \frac{-\tilde{J}}{2}\log(2\pi\sigma_v^2) - \sum_{j\in\tilde{J}} \frac{\mathbb{E}\left[(r_j - \gamma_0 - \gamma_1 x_j)^2\right]}{2\sigma_v^2}$$
$$+ \frac{-J}{2}\log(2\pi\sigma_\epsilon^2) - \sum_{j=1}^{J} \frac{\mathbb{E}\left[(x_j - x_{j-1})^2\right]}{2\sigma_\epsilon^2}.$$

Where $\tilde{J} = \{j|n_j = 1\}$ indicates the locations of neural impulses. The algorithm iterates between the E-step and the M-step until convergence.

1.2 Performance State Decoder

In the performance decoder, the E-step equations are slightly different than the arousal case. To decode the performance we use one binary and one continuous observation at trial k. A detail derivation of such decoder can be found in (Prerau et al., 2009). The forward filter equations are presented as,

Predict:

$$z_{k|k-1} = z_{k-1|k-1} \tag{S14}$$
$$s^2_{k|k-1} = s^2_{k-1|k-1} + \sigma^2_w$$

Update:

$$z_{k|k} = z_{k|k-1} + \frac{s^2_{k|k-1}}{\alpha_1^2 s^2_{k|k-1} + \sigma^2_\delta}\Bigg[\sigma^2_\delta(m_k - p_{k|k}) \tag{S15}$$

$$+ \alpha_1(I_k - \alpha_0 - \alpha_1 z_{k|k-1})\Bigg]$$

$$s^2_{k|k} = \left[\frac{1}{s^2_{k|k-1}} + p_{k|k}(1 - p_{k|k}) + \frac{\alpha_1^2}{\sigma^2_\delta}\right]^{-1} \tag{S16}$$

The smoother backward smoother can be derived as

$$B_k = \frac{s^2_{k|k}}{s^2_{k+1|k}}, \tag{S17}$$

$$z_{k|K} = z_{k|k} + B_k(z_{k+1|K} - z_{k+1|k}), \tag{S18}$$

$$s^2_{k|K} = s^2_{k|k} + B_k^2(s^2_{k+1|K} - s^2_{k+1|k}). \tag{S19}$$

In the M-step, the expected values of z_k^2, and $z_k z_{k-1}$ can be written as

$$\mathbb{E}[z_k^2] = z^2_{k|K} + s^2_{k|K}, \tag{S20}$$

$$\mathbb{E}[z_{k+1} z_k] = z_{k+1|K} z_{k|K} + B_k s^2_{k+1|K}; \tag{S21}$$

and the expected log-likelihood function $\mathbb{E}[Q_2]$ is noted as

$$\mathbb{E}[Q_2] = \sum_{k=1}^{K} \mathbb{E}[m_k(\mu + z_k) - \log(1 + e^{\mu + z_k})] \tag{S22}$$

$$+ \frac{-K}{2}\log(2\pi\sigma^2_\delta) - \sum_{k=1}^{K} \frac{\mathbb{E}\Big[(I_k - \alpha_0 - \alpha_1 z_k)^2\Big]}{2\sigma^2_\delta}$$

$$+ \frac{-K}{2}\log(2\pi\sigma^2_w) - \sum_{k=1}^{K} \frac{\mathbb{E}\Big[(z_k - z_{k-1})^2\Big]}{2\sigma^2_w}.$$

The unknown parameters can be found such that they maximize $\mathbb{E}[Q_2]$. The algorithm iterates between the E-step and the M-step until convergence.

2 SUPPLEMENTARY FIGURES

Figures S1 to S5 present the presents the epochs of oxygenated hemoglobin (HbO) concentration and performance across the task blocks for all the studied participants. The highest positive correlation can be seen within the 3-back task blocks for all of the participants:

- For participant 1, the highest HbO and performance correlation corresponds to the LB1 region during the 3-back task blocks within the caalming session.
- For participant 2, the highest HbO and performance correlation corresponds to the RF1 region during the 3-back task blocks within the exciting session.
- For participant 3, the highest HbO and performance correlation corresponds to the LF4 region during the 3-back task blocks within the exciting session.
- For participant 4, the highest HbO and performance correlation corresponds to the LF3 region during the 3-back task blocks within the calming session.
- For participant 5, the highest HbO and performance correlation corresponds to the LF2 region during the 3-back task blocks within the calming session.

Also, for all of the participants except for participant 2, the highest correlation would be associated with the left side of the brain. Considering the the prefrontal cortex (PFC) and occipital areas (OC) area, we can see that except for participant 1, the highest performance and HbO correlation presented within the studied PFC regions.

Looking into Figures S1 to S5, we can observe two characteristic trends in performance among the participants. First, the performance signal has the inverted-U shape through 1-back trials. Secondly, the lowest performance occurs at the third trial of the 3-back task block.

3 SUPPLEMENTARY DISCUSSION

The observed inverted-U shape performance through 1-back trials may be an indicator of the participant's engagement over time. In other words, there might be a possibility that at the beginning of the 1-back blocks, participants were not fully engaged in performing the task, and it could take time for participants to be fully engaged with the experiment. However, it should be noted the participants' performance start to fall after a while. This performance reduction may be an indicator of boredom. Specifically, the 1-back task is easy to perform, and over time, participants might fall out of the flow and experience boredom Murphy et al. (2014). It is important to highlight that by visual inspection, it can be seen that participants present a relatively earlier sharp performance reduction in the presence of calming background music. In contrast, the sharp performance reduction occurs later in the presence of exciting background music.

Another observed trend is the occurrence of the lowest performance at the third trial of the 3-back task block. This specific observation can be pretty complex and affected by a combination of factors such as arousal variability and its interplay with performance, cognitive overload at the third trial followed by unconscious or conscious strategy adjustment, or even random variation, given the small sample size.

The one point that needs to be highlighted is that the absence of linearly or exponentially increasing performance over the trials may be interpreted as the absence of learning or habituation effect in the n-back task Fekri Azgomi et al. (2023) while further investigations are needed to validate this point. To summarize, multiple plausible factors may be associated with the observed trends in performance. However, to identify the exact causes, further studies with a higher number of participants, a higher number of n-back difficulty levels, and the inclusion of a control group would be required.

REFERENCES

Fekri Azgomi, H., F. Branco, L. R., Amin, M. R., Khazaei, S., and Faghih, R. T. (2023). Regulation of brain cognitive states through auditory, gustatory, and olfactory stimulation with wearable monitoring. *Scientific reports* 13, 12399

Murphy, C., Chertoff, D., Guerrero, M., and Moffitt, K. (2014). Design better games: Flow, motivation, and fun. *Design and development of training games: Practical guidelines from a multidisciplinary perspective* , 1773

Prerau, M. J., Smith, A. C., Eden, U. T., Kubota, Y., Yanike, M., Suzuki, W., et al. (2009). Characterizing learning by simultaneous analysis of continuous and binary measures of performance. *Journal of neurophysiology* 102, 3060–3072

Wickramasuriya, D. S. and Faghih, R. T. (2020). A marked point process filtering approach for tracking sympathetic arousal from skin conductance. *IEEE Access* 8, 68499–68513

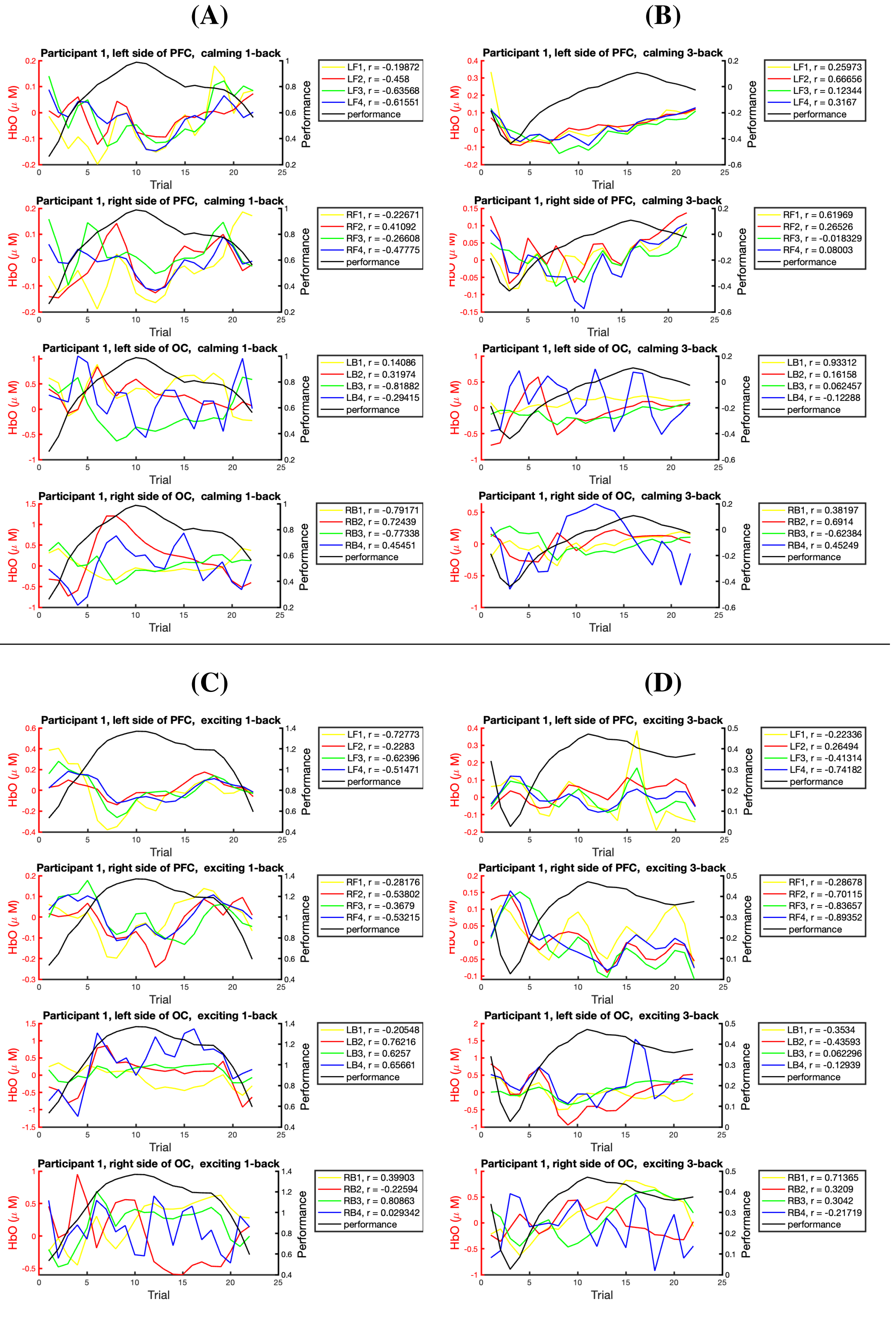

Figure S1. The correlation study for the epoch of HbO signal and performance state across the task blocks for participant 1. The sub-figures present: **(A)** the HbO and performance data recorded during the 1-back task blocks within the calming music. **(B)** the HbO and performance data recorded during the 3-back task blocks within the calming music. **(C)** the HbO and performance data recorded during the 1-back task blocks within the exciting music. **(D)** the HbO and performance data recorded during the 3-back task blocks within the exciting music. The sub-plots in each sub-figure, from top to bottom, represent: the HbO data collected from the left side of the PFC, right side of the PFC, left side of the OC area, and right side of the OC area, respectively. The legend box located on the right side of each subplot presents the Pearson correlation coefficients associated with the studied brain regions.

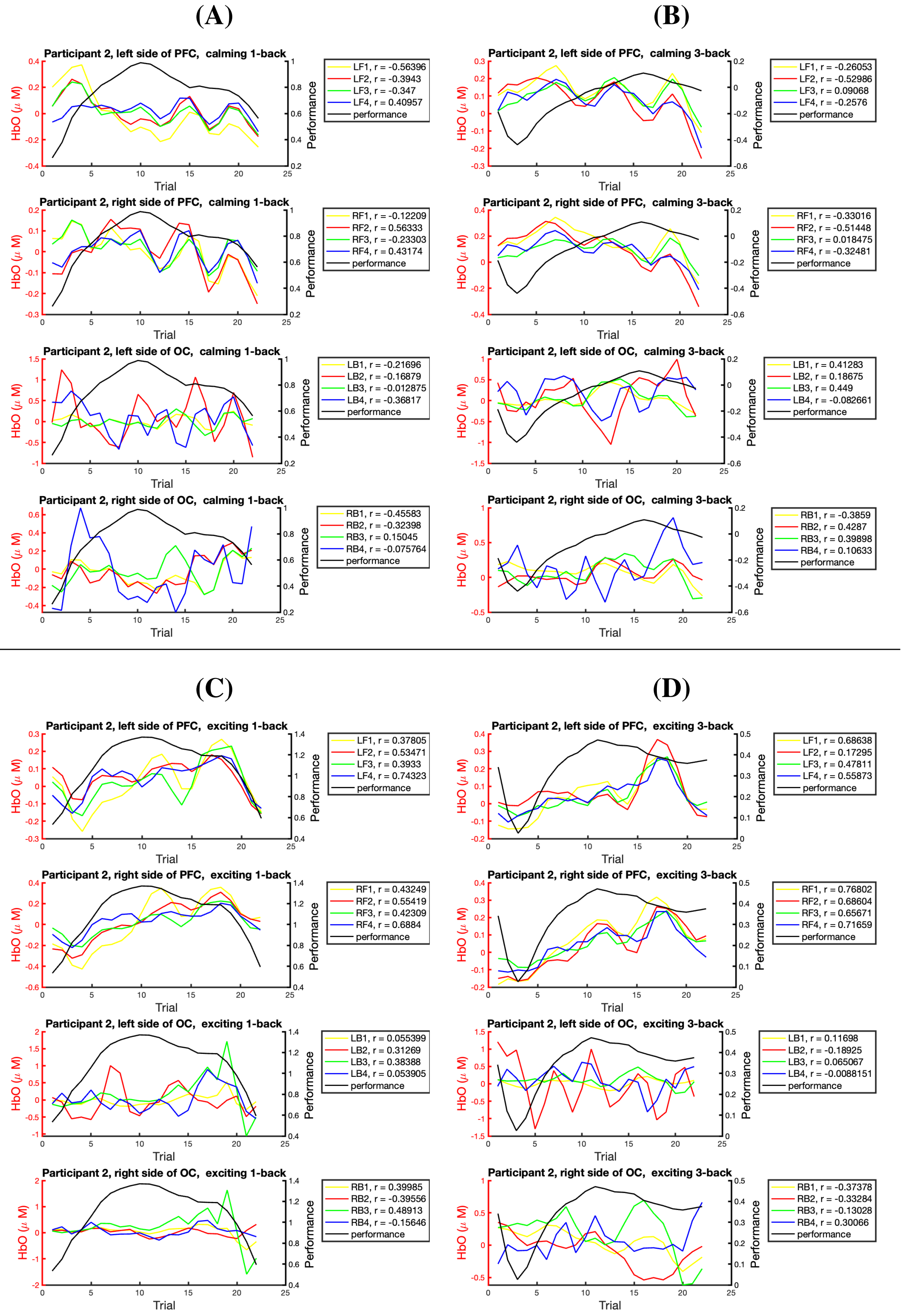

Figure S2. The correlation study for the epoch of HbO signal and performance state across the task blocks for participant 2. The sub-figures present: **(A)** the HbO and performance data recorded during the 1-back task blocks within the calming music. **(B)** the HbO and performance data recorded during the 3-back task blocks within the calming music. **(C)** the HbO and performance data recorded during the 1-back task blocks within the exciting music. **(D)** the HbO and performance data recorded during the 3-back task blocks within the exciting music. The sub-plots in each sub-figure, from top to bottom, represent: the HbO data collected from the left side of the PFC, right side of the PFC, left side of the OC area, and right side of the OC area, respectively. The legend box located on the right side of each subplot presents the Pearson correlation coefficients associated with the studied brain regions.

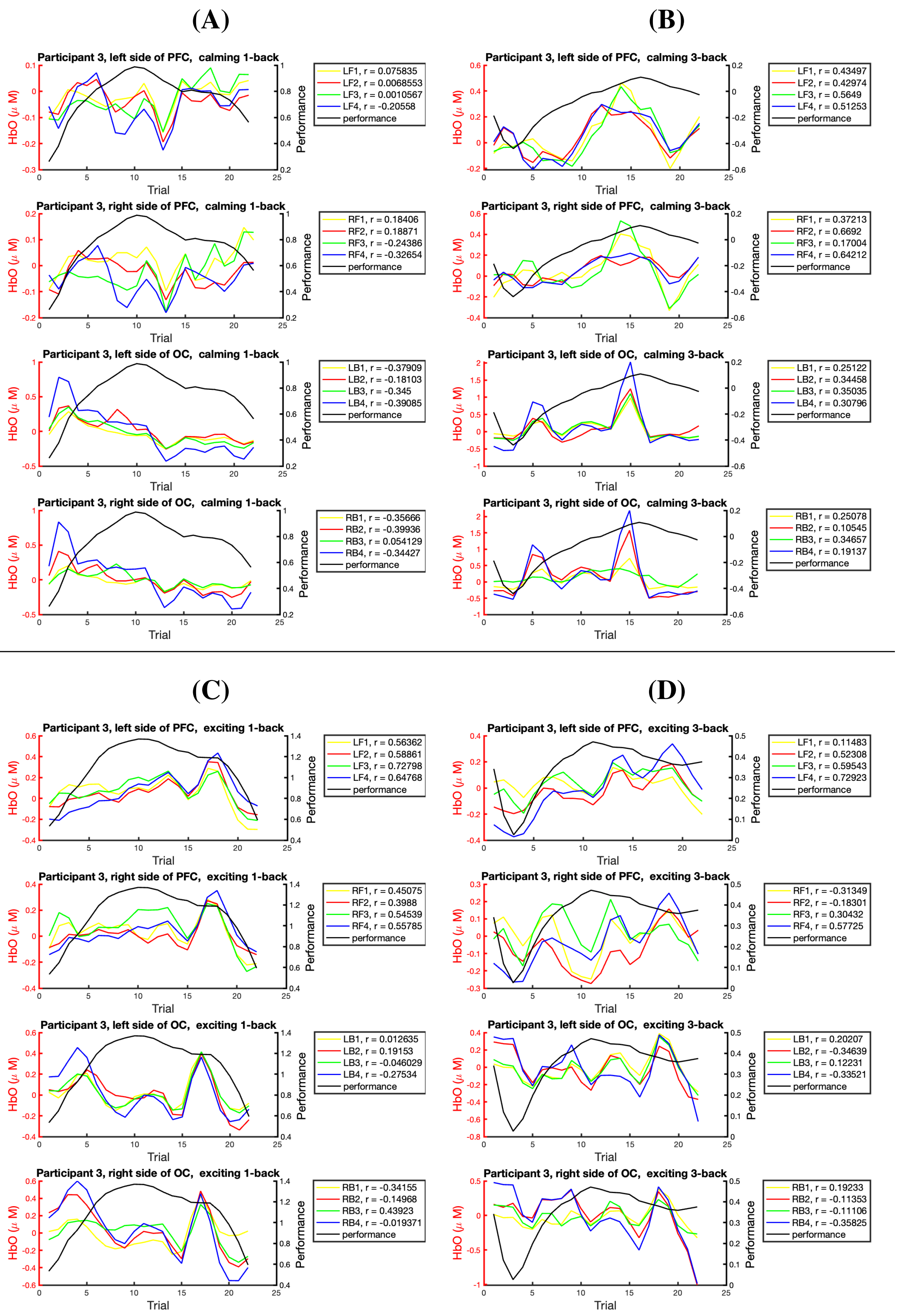

Figure S3. The correlation study for the epoch of HbO signal and performance state across the task blocks for participant 3. The sub-figures present: **(A)** the HbO and performance data recorded during the 1-back task blocks within the calming music. **(B)** the HbO and performance data recorded during the 3-back task blocks within the calming music. **(C)** the HbO and performance data recorded during the 1-back task blocks within the exciting music. **(D)** the HbO and performance data recorded during the 3-back task blocks within the exciting music. The sub-plots in each sub-figure, from top to bottom, represent: the HbO data collected from the left side of the PFC, right side of the PFC, left side of the OC area, and right side of the OC area, respectively. The legend box located on the right side of each subplot presents the Pearson correlation coefficients associated with the studied brain regions.

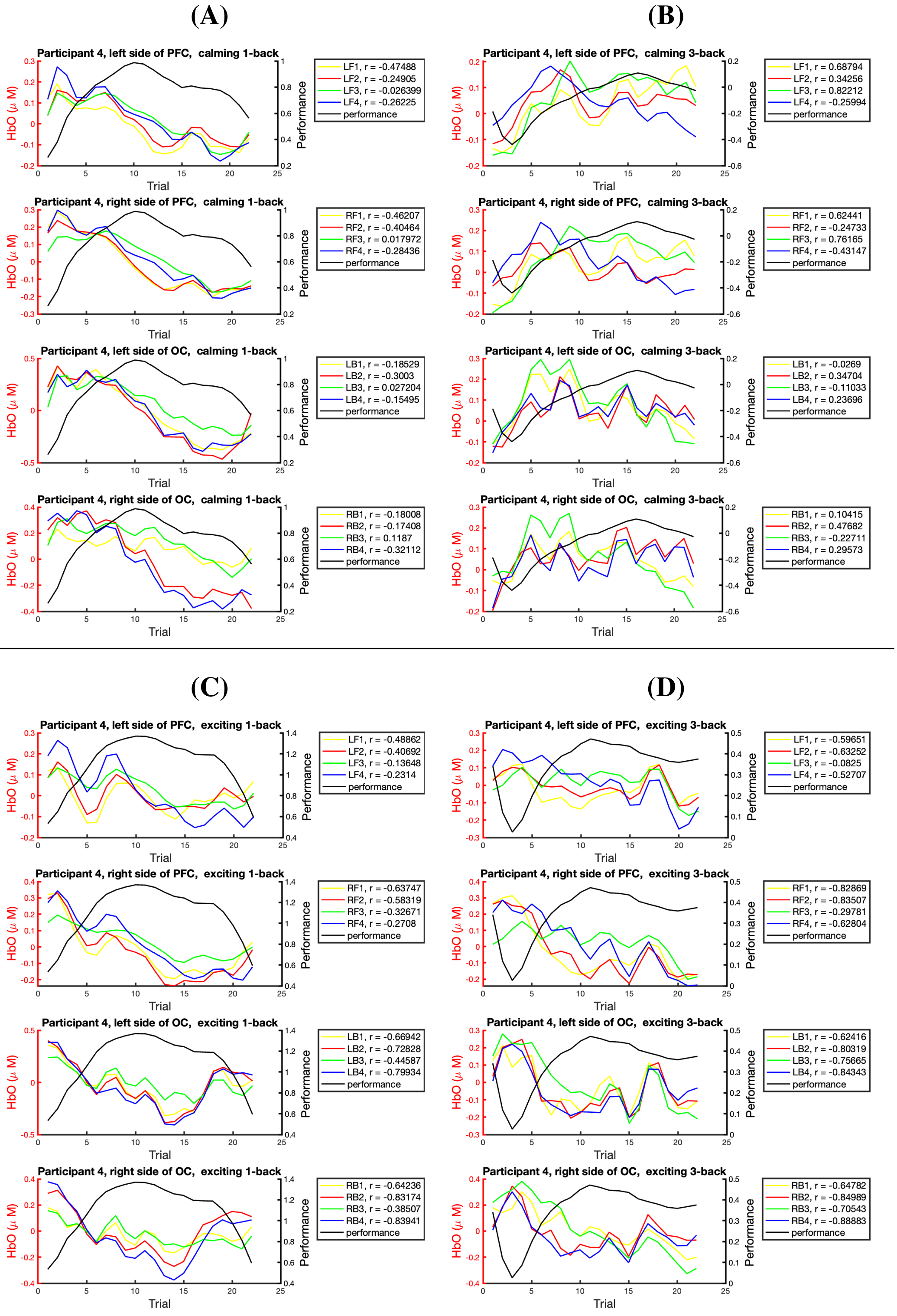

Figure S4. The correlation study for the epoch of HbO signal and performance state across the task blocks for participant 4. The sub-figures present: **(A)** the HbO and performance data recorded during the 1-back task blocks within the calming music. **(B)** the HbO and performance data recorded during the 3-back task blocks within the calming music. **(C)** the HbO and performance data recorded during the 1-back task blocks within the exciting music. **(D)** the HbO and performance data recorded during the 3-back task blocks within the exciting music. The sub-plots in each sub-figure, from top to bottom, represent: the HbO data collected from the left side of the PFC, right side of the PFC, left side of the OC area, and right side of the OC area, respectively. The legend box located on the right side of each subplot presents the Pearson correlation coefficients associated with the studied brain regions.

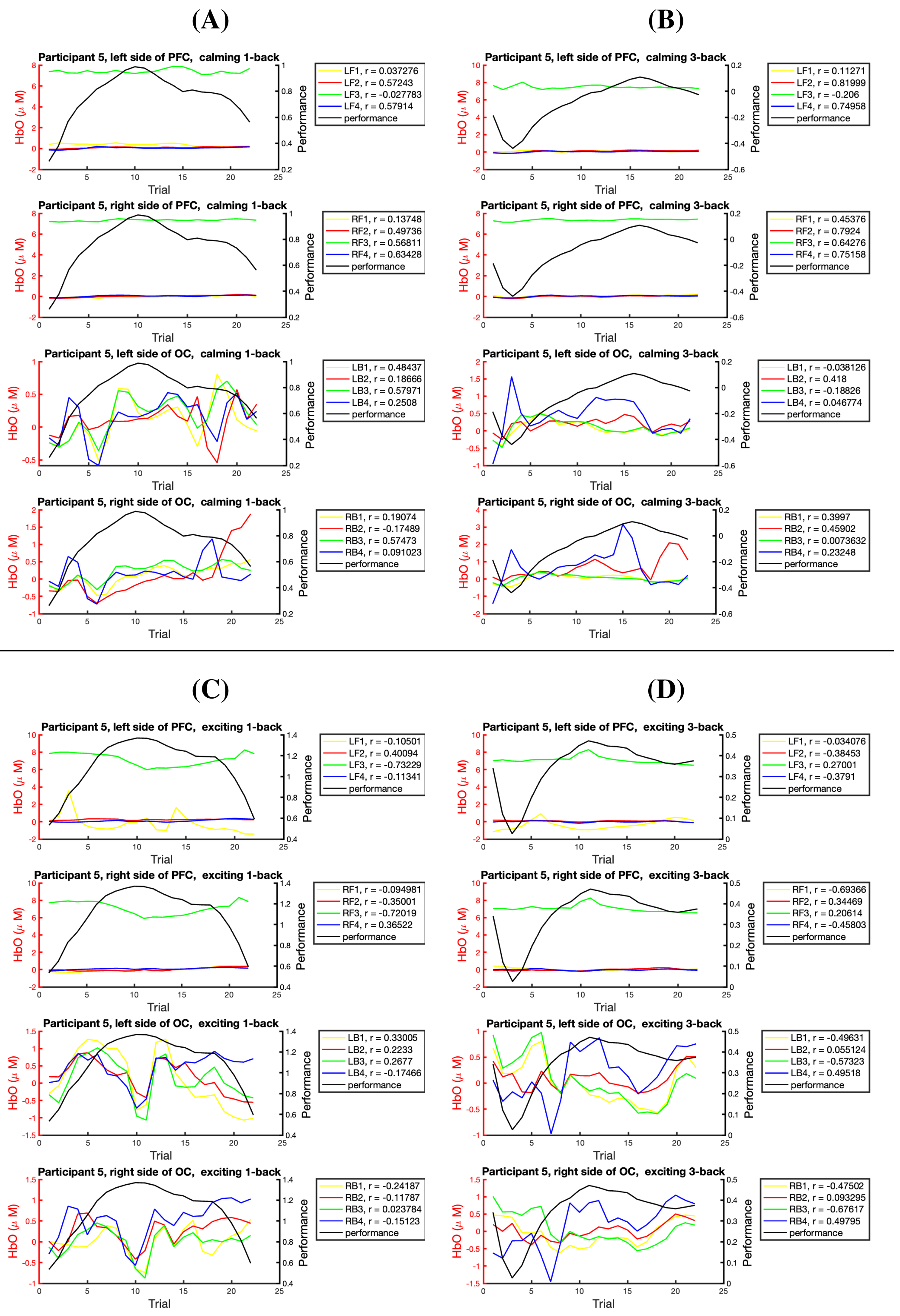

Figure S5. The correlation study for the epoch of HbO signal and performance state across the task blocks for participant 5. The sub-figures present: **(A)** the HbO and performance data recorded during the 1-back task blocks within the calming music. **(B)** the HbO and performance data recorded during the 3-back task blocks within the calming music. **(C)** the HbO and performance data recorded during the 1-back task blocks within the exciting music. **(D)** the HbO and performance data recorded during the 3-back task blocks within the exciting music. The sub-plots in each sub-figure, from top to bottom, represent: the HbO data collected from the left side of the PFC, right side of the PFC, left side of the OC area, and right side of the OC area, respectively. The legend box located on the right side of each subplot presents the Pearson correlation coefficients associated with the studied brain regions.
